# Proteomic Analysis of Lymphoblastoid Cells from Nasu-Hakola Patients: A Step Forward in Our Understanding of This Neurodegenerative Disorder

**DOI:** 10.1371/journal.pone.0110073

**Published:** 2014-12-03

**Authors:** Serena Giuliano, Anna Maria Agresta, Antonella De Palma, Simona Viglio, Pierluigi Mauri, Marco Fumagalli, Paolo Iadarola, Lorenza Montalbetti, Roberta Salvini, Anna Bardoni

**Affiliations:** 1 Department of Molecular Medicine, Biochemistry Unit, University of Pavia, Pavia, Italy; 2 Laboratoire d’excellence-Ion channel science and therapeutics, UMR, CNRS, Nice, France; 3 Institute for Biochemical Technologies, Proteomics and Metabolomics Unit, National Research Council, Segrate (Milano), Italy; 4 Department of Biology and Biotechnologies, Biochemistry Unit, University of Pavia, Pavia, Italy; 5 Department of Brain and Behavioral Sciences, University of Pavia, Pavia, Italy; Nathan Kline Institute and New York University School of Medicine, United States of America

## Abstract

Nasu-Hakola disease (NHD) is a recessively inherited rare disorder characterized by a combination of neuropsychiatric and bone symptoms which, while being unique to this disease, do not provide a rationale for the unambiguous identification of patients. These individuals, in fact, are likely to go unrecognized either because they are considered to be affected by other kinds of dementia or by fibrous dysplasia of bone. Given that dementia in NHD has much in common with Alzheimer’s disease and other neurodegenerative disorders, it cannot be expected to achieve the differential diagnosis of this disease without performing a genetic analysis. Under this scenario, the availability of protein biomarkers would indeed provide a novel context to facilitate interpretation of symptoms and to make the precise identification of this disease possible. The work here reported was designed to generate, for the first time, protein profiles of lymphoblastoid cells from NHD patients. Two-dimensional electrophoresis (2-DE) and nano liquid chromatography-tandem mass spectrometry (nLC-MS/MS) have been applied to all components of an Italian family (seven subjects) and to five healthy subjects included as controls. Comparative analyses revealed differences in the expression profile of 21 proteins involved in glucose metabolism and information pathways as well as in stress responses.

## Introduction

Nasu-Hakola disease (NHD) also referred to as Polycystic Lipomembranous Osteodysplasia with Sclerosing Leukoencephalopathy (PLOSL), is a recessively inherited rare disorder characterized by a combination of pre-senile frontal dementia and systemic bone cysts formation [1]–[3]. Formally, in the natural progression of this disorder the following four different stages may be identified: i) latent disease (asymptomatic); ii) bone implication (pathological fractures); iii) early neurological symptoms (patient’s personality changes and first dementia symptoms begin to arise) and iv) late neurological stage in which patients show symptoms of profound dementia and begin to lose their motility [4]. NHD has been demonstrated to rise from a structural defect in the DNAX-activating protein 12 gene (DAP12 gene, also called TYROBP, for tyrosine-kinase binding protein) or in the Triggering Receptor Expressed on Myeloid cells 2 (TREM 2) gene, the two genes encoding for different subunits of the same membrane receptor signaling complex [5], [6]. Investigations on the role of DAP12 in B cells have been carried out by Nakano-Yokomizo *et al.* [7] by generating DAP12-deficient mouse B cells. Based on their results, this gene was found to play an important role in antigen-specific immune responses by B cells *in vivo* [7]. Despite this conclusion, the patho-physiological significance of the DAP12 involvement in humoral immune responses remains uncertain and further studies are needed to gain insights into these mechanisms. While the signaling pathways involved in DAP12-mediated inhibition have not been completely understood yet [8], [9], the relationship between inflammation and neuro-degeneration for a number of disorders, including Multiple Sclerosis (MS); Alzheimer’s Disease (AD) and Parkinson’s Disease (PD), is gradually emerging [10]. Taken together, these data provide a new and larger context for hypothesizing that a variety of mechanisms involved in immune system may contribute to neuronal damage [10]–[12]. In this respect, TREM2 gene is known to possess an immunoglobulin superfamily domain [13] and to be expressed in peripheral blood cells such as macrophage- and monocyte-derived dendritic cells. This gene plays important roles in innate and adaptive immunity [14] and is most likely involved in chronic inflammatory diseases [15], [16]. This hypothesis has been recently confirmed by Paloneva *et al.* [6] who postulated the involvement of TREM2 in chronic inflammatory disorders of central nervous system (CNS).

It was in the course of NHD investigations on patients (belonging to an Italian family) negative for mutations in DAP12 gene, that a conversion of nucleotide C to T (that determines the change of Gln 33 to a stop codon (Q33X) at position 97 in exon 2 of TREM2 gene, was shown to be responsible for the disease [17]. Interestingly, apparently identical clinical phenotype has been observed in both patients with TREM2- and DAP12-mutations [17], [18]. Moreover, microglial TREM2 was shown to be involved in phagocytosis of apoptotic cellular material [19], [20], a function which is essential to keep central nervous system homeostasis. Thus, it seems plausible to state that a nonfunctional TREM2 could play a pivotal role in brain damage, due to the accumulation of toxic products such as apoptotic material.

Despite the worldwide distribution of NHD, Finland and Japan are the countries with the highest number of cases so far reported [3], [16]. Outside these countries the disease is unknown and/or underestimated [18]. While the combination of neuropsychiatric and bone symptoms is unique to this disease, they do not provide a rationale for the unambiguous identification of patients. Patients in fact are likely to go unrecognized either because they are considered to be affected by other kinds of dementia or by fibrous dysplasia of bone [18]. As far as the clinical characteristics are concerned, dementia has much in common with Alzheimer’s or Pick’s diseases [3] and, despite the existence of peculiar symptoms, it cannot be expected to obtain the differential diagnosis of NHD without performing a genetic analysis. Under this scenario, it appears clear that the availability of protein biomarkers would provide a novel context for the precise identification of Nasu-Hakola disease thus greatly enhancing the interpretation of symptoms.

The means of analyzing this biological signaling have undergone dramatic changes over the last few years. Not that long time ago, the procedures traditionally in use allowed to identify proteins one by one. The great advances in technologies and experimental strategies, mainly in the field of proteomics (and genomics), have enabled a general shift in paradigm from dedicating work to the analysis of a single protein to the analysis of biochemical (and cellular) processes. In particular, two-dimensional gel electrophoresis (2-DE) techniques have been implemented and gained high popularity in the search of over- or under-expressed proteins in pathological conditions [11], [21]–[23]. In this respect, to provide insights into the molecular mechanisms of Nasu-Hakola disease, 2-DE proteomic profiles of lymphoblastoid cells from seven individuals (six patients and one healthy subject), all belonging to an Italian family, have been generated for the first time. The comparison of these with proteomic patterns obtained from five additional controls aimed at identifying differentially-expressed proteins that could be candidate biomarkers of the disease. Proteins were identifed by nano Liquid Chromatography-tandem Mass Spectrometry (nLC-MS/MS) and validated by western blotting analysis.

The current report contains an accurate description of this comprehensive study.

## Materials and Methods

### Reagents

REDtaq Genomic DNA polymerase was from Sigma Aldrich (St. Louis, MO, USA). Carrier ampholytes and immobilized pH gradient gel strips were from GE Healthcare (Uppsala, Sweden). *RC DC* Protein Assay Kit was purchased from BioRad (Richmond, CA, USA). Antibodies used to validate a good number of proteins identified were from Santa Cruz Biotechnology (Dallas, TX, USA). All other reagents were of analytical grade and used without further purification.

### Subjects

This investigation was performed on twelve subjects in total. Seven individuals belonged to the same Italian family and consisted of two patients with homozygous C-to-T mutation at position 97 in exon 2 of TREM2 gene; four patients with heterozygous mutation, and one healthy individual (control). Five additional healthy subjects (female volunteers from the laboratory) participated to this study as controls. At the moment of blood withdrawal, heterozygotes (He) for the mutated allele showed impairment of visuo-spatial memory and mild hypoperfusion in the right basal ganglia. Homozygotes (Ho), in the first stage of the disease, presented the same neuropsychological and neuro-functional patterns. In the subsequent stages, however, neuropsychological tests were no longer administrable and the hyperfusion became severe and diffuse. Obviously, the homozygote subject for the wild-type allele (wt) presented normal neuropsychological and neuro-imaging findings. Additional information about clinical conditions of these subjects may be found in reference [18].

### Samples

Individuals considered in this study had been previously involved in the clinical investigation described in reference [17]. At the moment of blood withdrawal all of them signed a written informed consent that was approved by the Ethics Committee of the Neurological Institute “C.Mondino”, Pavia and the “Laura Fossati Foundation”, Montesegale, Pavia, who reviewed and authorized studies on these patients. Frozen blood samples (20 mL) were thawed and treated as follows: an aliquot (10 mL) was centrifuged at 1100 × g for 10 min to separate the plasma. Another aliquot (10 mL) was used to isolate the B-lymphocytes that were immortalized by treatment with Epstein-Barr Virus (EBV) [24]. The lymphoblastoid B-cell lines were maintained in suspension culture in RPMI 1640 medium supplemented with 10% fetal bovine serum, 4 mM glutamine, streptomycin, and penicillin. To obtain total extracts, cells were harvested by centrifugation (1300 × *g* for 5 min at 4°C); re-suspended in 8M urea, 4% (w/v) CHAPS, 65 mM DTE in the presence of a protease inhibitors cocktail (Sigma Aldrich) and finally sonicated three consecutive times (for 5 s) at 20 kHz. Protein concentration was determined using the *RC DC* (reducing agent and detergent compatible) Protein Assay Kit, with BSA as standard.

### Purification and reverse transcription of total cellular RNA and PCR amplification

The total lymphoblastoid B-cell RNA was purified by using the RNeasy mini kit (Qiagen, Manchester, UK), according to the instructions of the manufacturer. The concentration of cellular RNA was quantified by determining the optical density at 260 nm. Total cellular RNA was reverse-transcripted by means of the RNA PCR Core Kit (Applied Biosystems, Foster City, CA, USA). The resulting complementary DNA (cDNA, 200 ng) was submitted to conventional PCR amplification in a 25 µl reaction volume on a PCR SPRINT Thermal Cycler (Thermo Electron Corporation, MA, USA). The parameters used for PCR amplification were the following: i) initial DNA denaturation at 94°C for 3 min followed by 35 cycles of denaturation at 94°C for 30 s; ii) primer annealing at 57°C for 30 s, and elongation at 72°C for 30 s; iii) extension at 72°C for 3 min. Detection of the PCR amplification products was performed using the PCR 5′-TCT TTG TCA CAG AGC TGT CC-3′ (sense) and 5′-AGG GTA TCG TCT GTG ATG GC-3′ (antisense) primers (PRIMM Co. Ltd, Milan, Italy). An aliquot (10 µl) of each PCR reaction sample was finally submitted to 1.5% agarose gel electrophoresis to visualize the products by staining with ethidium bromide.

### Two-dimensional Gel Electrophoresis (2-DE)

Protein extracts were loaded on nonlinear (NL) pH 3–10 gradient range IPG gel strips (18 cm length). One of the pretreatment procedures consisted in rehydrating gel strips in a buffer containing 8 M urea, 4% (w/v) CHAPS, 65 mM DTE, 0.8% (v/v) carrier ampholytes and traces of bromophenol blue. Rehydration was performed for 8 h at 16°C using a voltage of 30 V. The same voltage regime was applied for each step of isoelectrofocusing (IEF) both in case of nonlinear pH 3–10 or linear pH 4–7 IPG strips according to a program driven by the BioRad (CA, USA) Ettan IPGphor system (1 h at 120 V; 30 min at 300 V; linear ramping from 300 to 3500 V in 3 h; 10 min at 5000 V and then 7950 V to reach a total of 62 KV/h). Reduction/alkylation steps were applied between the first and the second dimension. The focused IPG strips were incubated for 12 min at room temperature in 6 M urea, 2% (w/v) SDS, 50 mM Tris pH 6.8, glycerol 30% containing 2% (w/v) DTE and for 5 min in an equilibration buffer containing 2.5% (w/v) iodoacetamide.

At the end of the IEF step, strips were hold in place with 0.4% low melting temperature agarose and loaded onto a 20×18 cm slab, 9–16% SDS-polyacrylamide gels. Electrophoresis was carried out at a constant current of 40 mA per gel in a PROTEAN II xi 2-D Cell (Bio-Rad) equipment. The 2-DE gels were stained with “Blue silver” (colloidal Coomassie G-250 staining), according to Candiano *et al.* [25]. Digital images of stained gels were acquired using VersaDoc Imaging Model 3000 (BioRad) and then subjected to quali/quantitative analysis using the PD Quest (BioRad) version 8.0.1 software. Scanned images were filtered and smoothed to remove background noise; vertical/horizontal streaking; gel artifacts and then normalized to eliminate the variability of each sample. The software then determined the amount of spots present and calculated their intensity by applying the following algorithm: peak value (ODs/image units) *σ_x_ *σ_y_ (standard deviations in x and y).

### Protein identification

Proteins were identified using the following approaches: i) by comparing our maps with the SWISS-2D PAGE (http://www.expasy.org/sprot) map, ii) by applying nano liquid chromatography-tandem mass spectrometry (nLC-MS/MS) to the spots excised from the maps obtained in this study and treated as indicated below; and iii) by comparing our maps with previously published lymphocyte maps [26], [27].

### Protein validation

Differentially-expressed proteins were transferred onto Millipore PVDF membranes (Billerica, MA, USA) at 200 mA for 1.20 h in transfer buffer (25 mM Tris, 192 mM glycine, pH = 8.3, containing 20% methanol). Membranes were blocked in 5% of non-fat dry milk in PBS-buffer for 1 h at room temperature on rollerbank and were incubated overnight at 4°C in 1% non-fat dry milk in PBS-buffer, containing 0.05% Tween-20, in the presence of primary antibodies (goat anti-human vimentin, actin, glyceraldheyde 3-P dehydrogenase, heterogeneous nuclear ribonucleoprotein, heat shock protein 70, alcohol dehydrogenase NADP, phosphoglycerate kinase 1, ubiquitin carboxy-terminal hydrolase L1 and elongation factor 1) diluted 1∶5000. Subsequently, they were washed in PBS-buffer containing 0.05% Tween-20 and reacted for 1 h with the goat anti-rabbit Ig secondary antibody conjugated with HRP diluted 1∶5000. After washing in the same buffer indicated above, membranes were incubated for 2 min in 6 ml of ECL Advance Western Blotting Detection Kit (GE-Healthcare) and finally proteins visualized using ImageQuantTM LAS 4000 mini Biomolecular Imager (GE Healthcare).

### Reproducibility of the study

To verify the reproducibility of the study, 2-DE maps were carried out in triplicate for each subject. A total of 36 gels was thus produced. Those presented in this report are the best representative gels among all generated that showed spots constantly present. The spot averages of the replicated gels from each repeated operation were used for calculating the mean ± SD spot number. Experimental steps concerning sample preparation; electrophoresis run and gel staining were performed “in parallel” on all samples.

### LC-MS/MS analysis

#### In-gel digestion

The selected spots, excised from 2D-gel and chopped into smaller pieces, were incubated on a shaking thermo block at 30°C for 15 min with wash solution (50∶50 v/v CH_3_CN: 0.1 M NH_4_HCO_3_, pH 8.0). This procedure was repeated until complete destaining. After removal (under vacuum at 60°C for 10 min) of the wash solution, gel pieces were resuspended in 30 µL of 0.1 M NH_4_HCO_3_ pH 8.0 and digested overnight at 37°C by addition of 0.5 µg sequencing grade trypsin (Promega, Madison, WI, USA). Peptides were then extracted sequentially from gel matrix by treatment (at 37°C for 15 min) with 30 µL of 50% CH_3_CN in water, 0.1% HCOOH and finally with 50 µL of 100% CH_3_CN. Each extraction involved 10 min of stirring followed by centrifugation and removal of the supernatant. The original supernatant and those obtained from sequential extractions were combined and dried. At the moment of use the peptide mixture was solubilized in 10 µL of 0.1% HCOOH for MS analyses.

#### Liquid Chromatography

Samples were analyzed using the Eksigent nanoLC-Ultra 2D System (Eksigent, part of AB SCIEX Dublin, CA, USA) combined with cHiPLC-nanoflex system (Eksigent) in trap-elute mode. Briefly, samples were first loaded on the cHiPLC trap (200 µm×500 µm ChromXP C18-CL, 3 µm, 120 Å) and washed in isocratic mode with 0.1% aqueous formic acid for 10 min at a flow rate of 3 µL/min. The automatic switching of cHiPLC ten-port valve then eluted the trapped mixture on a nano cHiPLC column (75 µm×15 cm ChromXP C18-CL, 3 µm, 120 Å), through a 40 min gradient of 5–60% acetonitrile (containing 0.1% formic acid), at a flow rate of 300 nL/min. To preserve system stability, in terms of elution times of components, trap and column were maintained at 35°C.

#### Mass Spectrometry

All experiments were acquired using a QExactive mass spectrometer (Thermo Fisher Scientific, San Josè, CA, USA), equipped with a nanospray ionization source (Thermo Fisher). Nanospray was achieved using a coated fused silica emitter (New Objective, Woburn, MA, USA) (360 µm o.d./50 µm i.d.; 730 µm tip i.d.) held at 1.5 kV. The ion transfer capillary was held at 220°C. Full mass spectra were recorded in positive ion mode over a 400–1600 m/z range and with a resolution setting of 70000 FWHM (@ m/z 200) with 1 microscan per second. Each full scan was followed by 7 MS/MS events, acquired at a resolution of 17,500 FWHM, sequentially generated in a data dependent manner on the top seven most abundant isotope patterns with charge ≥2, selected with an isolation window of 2 m/z for the survey scan, fragmented by higher energy collisional dissociation (HCD) with normalized collision energies of 30 and dynamically excluded for 30 s. The maximum ion injection times for the survey scan and the MS/MS scans were 50 and 200 ms and the ion target values were set at 10^6^ and 10^5^, respectively.

### Data Analysis

All data generated were searched using the Sequest search engine contained in the Thermo Scientific Proteome Discoverer software, version 1.4. The experimental MS/MS spectra were correlated to tryptic peptide sequences by comparison with the theoretical mass spectra obtained by *in silico* digestion of the human protein database (about 228763 entries), downloaded January 2013 from the National Centre for Biotechnology Information (NCBI) website (www.ncbi.nlm.nih.gov). The following criteria were used for the identification of peptide sequences and related proteins: trypsin as enzyme; three missed cleavages per peptide were allowed and mass tolerances of ±50 ppm for precursor ions and ±0.8 Da for fragment ions were used. Validation based on separate target and decoy searches and subsequent calculation of classical score-based false discovery rates (FDR) were used for assessing the statistical significance of the identifications. Finally, to assign a final score to proteins, the SEQUEST output data were filtered as follows: 1,5; 2.0; 2.25 and 2.5 were chosen as minimum values of correlation score (Xcorr) for single-; double-; triple- and quadrupole-charged ions, respectively. Only peptides with high confidence were considered; the protein grouping was enabled and the consensus score was set higher than 10.

## Results

### TREM2 expression

TREM-2 belongs to the immunoglobulin superfamily (Ig-SF) and, in the CNS, it is expressed in close association with DAP12 in microglial cells of frontal, temporal, parietal and basal ganglia, cerebellum and spinal cord. Although not all TREM2/DAP12 functions have been fully elucidated so far, NHD might be an interesting example of how primary microglial dysfunction can damage the CNS thus emerging as the prototype of a primary microglial disorder of the CNS. In light of this, microglia would have been expected to be the source of proteins for our research. However, due to obvious ethical considerations, access to these brain cells from living subjects under investigation was practically not available. To overcome this limitation, lymphobastoid B-cells have been used as a valid alternative to microglia. The rationale for this choice was the similarity in the expression of certain genes between lymphobastoid cells and microglia. In fact, although microglia and lymphocytes B do not share the same progenitor (they are generated from common myeloid progenitor and common lymphoid progenitor, respectively), both are antigen-presenting-cells (APC) and both express TREM2. TREM 2 expression in lymphocytes B had never been shown before and was the preliminary step of this work. Thus, cellular RNA from each sample was reverse transcripted and the resulting complementary DNA was submitted to conventional PCR amplification. An aliquot of each PCR reaction sample was then loaded on 1.5% agarose gel electrophoresis to visualize the products by staining with ethidium bromide. As shown in Fig. S1, the appearance of a clear band (see lanes 2 to 8, in which samples from wild type homozygote II3, representative of all controls, and all patients have been loaded) in correspondence of the 250 bp DNA ladder (lane 9) and the positive control band (Hela cells, lane 1), confirmed the presence of this protein in Lymphoblastoid B-cells.

**Figure 1:**
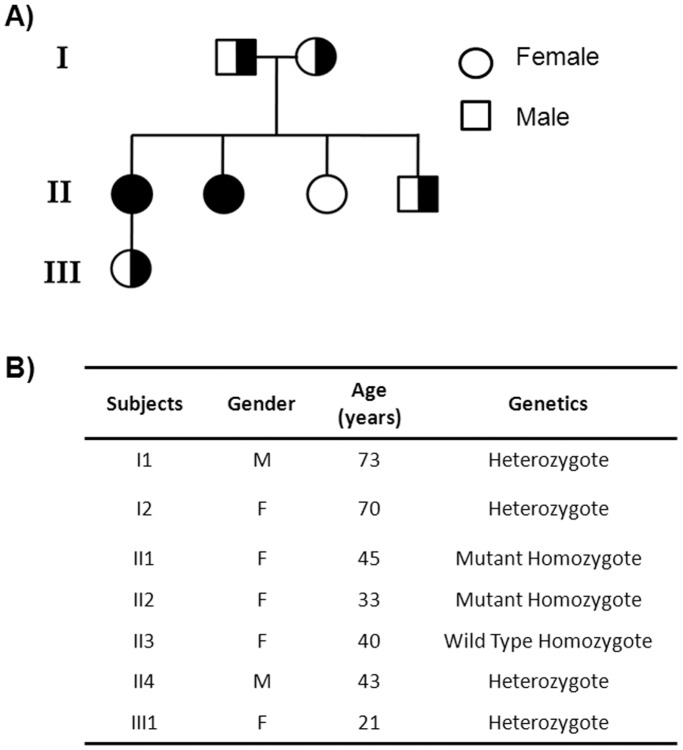
Pedigree of the Italian family considered in this study (Panel A). Demographic table showing the characteristics of subjects investigated (Panel B).

Based on these data, the proteomic profiles of lymphoblastoid B-cells from an entire Italian family made of seven components (six NHD patients and one healthy individual) have been performed. Five additional healthy subjects were analyzed as controls. The family pedigree and the demographic data of these individuals, which include gender; age and phenotype, are shown in panels A and B, respectively, of Figure 1.

### Two-dimensional map of Lymphoblastoid-B cells proteins

**Figure 2:**
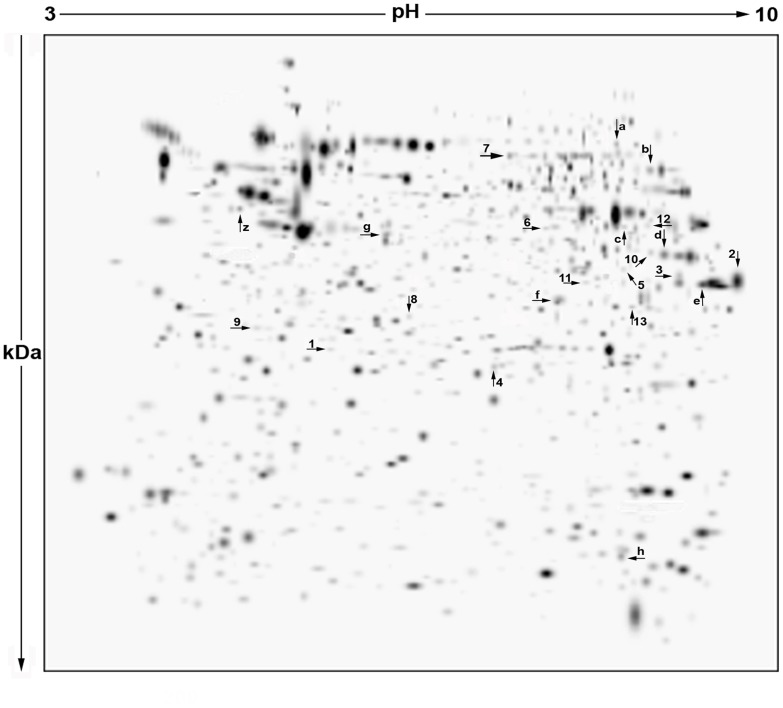
Two-dimensional electrophoretic map of proteins in Lymphoblastoid B-cells from individuals belonging to the family described in **Figure 1**. The virtual image reported here (Higher Master Gel, HMG), results from the correlation of master gels from each group of subjects and contains all the common and uncommon spots among groups of comparison. Spots differentially expressed among groups of subjects have been labeled by arrows with letters and numbers. Proteins contained in altered spots indicated by letters (**a** to **h**) were identified by gel-matching; those in spots arbitrarily indicated by numbers (1 to 13), by LC-MS/MS. For additional experimental details, see the text.

2-DE analyses of Lymphoblastoid B-cells from each of the twelve individuals were performed in triplicate. The three gels for each single subject were scanned and interpreted with the software indicated in the experimental section. The next step was the creation of a “Match Set” to compare all gels of a single group and to match the spots present. By using this match set, a synthetic image (Master Gel) was created that contained qualitative and quantitative data relative to all spots. The master gels from each group (wild type, wt; heterozygotes, He, and homozygotes, Ho) showed such a high similarity between protein patterns that they could easily be matched to each other. This facilitated the correlation of gels and the creation of a Higher Match Set and a virtual image indicated as Higher Master Gel (HMG) that contained all the common and uncommon spots between groups of comparison. This Higher Match Set allowed to determine the presence or absence of spots and the intensity values of common ones that could be submitted to statistical analysis. This approach showed a high level of reproducibility inside each group; typically, a mean of 907±41 protein spots were detected in coomassie stained gels of the controls; 886±38 for heterozygotes and 897±39 for homozygotes. The HMG image, comprehensive of all matched spots derived from master gels of three groups analyzed, is shown in Figure 2.

### Differentially expressed proteins

Spot quantities of all gels were normalized to remove non-expression related variations in spot intensity and data were exported as clipboard for further statistical analysis. The raw amount of each protein in a gel was divided by the total quantity of all proteins (spots) that were included in that gel. The results were evaluated in terms of spot optical density (OD). Statistical analysis of PDQuest data allowed to assess differences in protein abundance on a protein-by-protein basis. According to guidelines for differential proteomic research^27^, only spots that showed a change in density at p<0.05 (by nonparametric Wilcoxon test) among groups were considered to be “differentially expressed” in the three groups of subjects. This term was used here in the sense of differential protein abundance determined by several processes including changes in protein biosynthesis and modification or degradation. By using these criteria, 21 spots (indicated by arrows with numbers and letters in HMG of Figure 2) out of the 896±32 spots, differed by the ratio indicated above and were selected by the statistical program as spots having significant changes in intensity between lymphoblastoid B-cells of He/Ho and wt cells. All of them were common to the three groups of subjects investigated.

**Figure 3:**
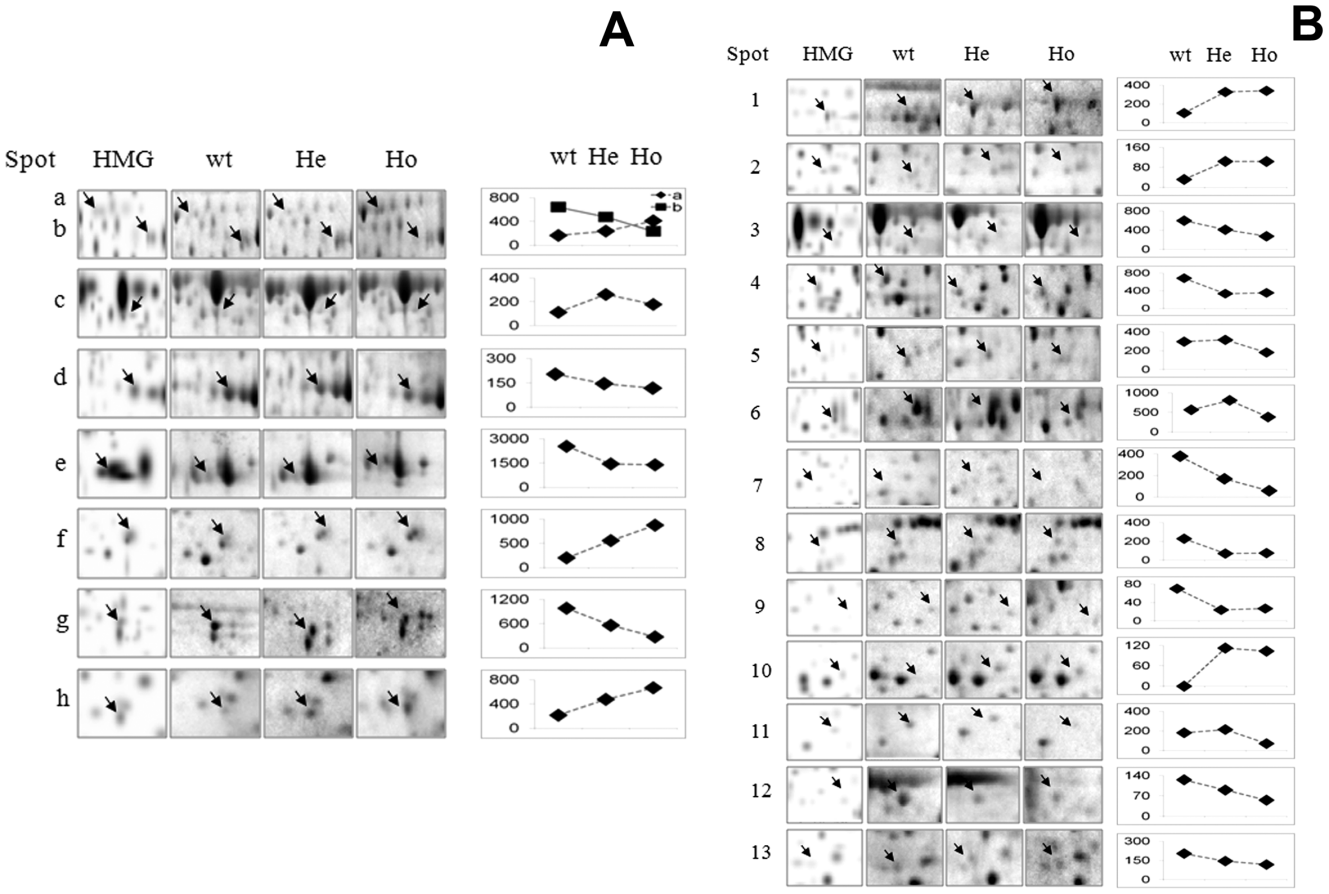
The zoomed region of each real stained gel in which altered spot (indicated by an arrow) is positioned, is shown in this figure. A single magnified gel view (representative of all others) for each group was horizontally aligned to highlight differences in spot density among groups. Aside of each horizontal set of panels, a graphical representation of changes was also reported. Spots **a** to **h** are shown in panel A; spots 1 to 13 are shown in panel B.

To highlight differences in spot density among groups, the region of each real stained gel in which spot of interest (indicated by an arrow) is positioned, was zoomed. By horizontally aligning a single magnified gel view (representative of all others) for each group, the set of panels shown in Figure 3 (panels A and B) was generated. For a better visual inspection of spot density variances for each individual, a graphical representation was reported aside of each horizontal set of panels.

Identification of proteins under altered spots was obviously the only way to answer the immediate question of whether these spots could contain any potential diagnostic biomarker of NHD.

Proteins under 8 spots indicated by letters **a** to **h** were identified by gel-matching while those under spots arbitrarily indicated by numbers 1 to 13, by LC-MS.

### Identification of proteins in spots a–h

The good matching (in terms of pI and M_r_ values) between spots **a** to **h** in our map of Figure 2 and spots observed in previously published human skin fibroblast maps, whose protein content was identified [26], [27], allowed to assign tentatively the identity to these proteins. This resulted in the list of proteins shown in Table 1.

**Table 1:** List of proteins identified by Gel Matching.

Spot	accession	Protein Name	MW Theoretical/Experimental*	pI Theoretical/Experimental*
**a**	21264428	Heat-Shock Protein 70	74/75–76	5,9/6.8
**b**	130348	Phosphoglycerate mutase 1	29/32	6,7/7.2
**c**	119339	Alpha-enolase	37/44–45	5,9/7.0
**d**	113606	Fructose-bisphosphate aldolase A	39/41–42	8,4/7.6
**e**	120649	Glyceraldehyde-3-phosphatedehydrogenase	36/33–34	8.6/8.4
**f**	44888310	Pyridoxal phosphate phosphatase	32/29–30	6.1/6.9
**g**	46397333	β Actin	42/44–45	5.3/5.6
**h**	1706278	Cystatin-B	11/10–11	6.9/7.2

*Experimental values have been determined from the mobility of protein spots.

### Identification of proteins in spots 1–13

The poor matching of spots 1–13 in Figure 2 with those of maps indicated above forced us to assign their identity by LC-MS/MS. Spots were carefully excised from the gel; destained; digested with trypsin and peptide mixtures submitted to nanoLC-MS/MS following the procedure detailed in the experimental section. After searching the MS fragmentation data against the databases indicated, a confident identification was obtained for all of the queried proteins. Unambiguous identification was achieved also in cases in which spot of interest (i.e. spot 6) contained small amounts of interfering components which co-migrated with major protein. For each protein identified, detailed identification data, including protein and gene names, accession number, uniprot ID, pI and molecular mass (theoretical); sequest score; spectral count and unique peptides identified; are shown in Table 2. Additional information concerning the primary sequence of all peptides identified for each of these proteins is shown in Table S1.

**Table 2:** List of proteins identified by LC-MS/MS.

SpotNo.	Protein name	Genename	GIAccession	UniProt ID	pI	Thr.MW (kDa)	Score	SpC	Peptides	UniquePeptides
**1**	Ubiquitin carboxy-terminal hydrolase L1	UCHL1	4185720	P09936	5,45	23,01	52,00	11	2	2
**2**	T-complex protein 1 subunit ε	CCT5	194381764	P48643	8,50	32,03	46,68	12	2	2
**3**	Δ(3,5)- Δ(2,4)-dienoyl-CoAisomerase, mitochondrial precursor	ECH1	70995211	Q13011	8,00	35,79	50,49	11	6	5
**4**	L-isoaspartyl/D-aspartyl O-methyltransferase	PCMT1	180637	P22061	6,52	24,70	57,37	13	1	1
**5**	Coproporphyrinogen oxidase	CPOX	433888	P36551	7,12	40,28	246,17	57	7	7
**6**	Heterogeneous nuclearribonucleoprotein H	HNRNPH1	48145673	P31943	6,18	49,10	171,58	37	6	6
**7**	T-complex protein 1 subunit γ	CCT3	63162572	P49368	6,49	60,50	74,73	17	5	5
**8**	Density-regulated protein	DENR	4755083	O43583	5,96	26,50	57,81	12	3	3
**9**	Elongation factor 1 **δ**	EEF1D	38522	P29692	5,06	31,20	30,34	5	1	1
**10**	Complex intermediate-associatedprotein 30, mitochondrial	NDUFAF1	49574510	Q9Y375	7,64	37,71	10,54	2	1	1
**11**	Alcohol dehydrogenase [NADP(+)]	AKR1A1	5174391	P14550	6,79	36,50	235,96	50	4	4
**12**	Phosphoglycerate kinase 1	PGK1	4505763	P00558	8,10	44,60	68,27	14	6	6
**13**	Voltage-dependent anion-selectivechannel protein 2	VDAC2	48146045	P45880	7,20	30,40	37,72	7	2	2

### Validation of proteins identified

To achieve the unambiguous identification of proteins under spots **a** to **h** and to answer the question about robustness of interpretation, gel spots were excised and submitted to the procedure indicated above. Not surprisingly, the results (shown in Table S2) confirmed the correct attribution of proteins. Our data, while suggesting that the process of spot assignment was unambiguous, indicated that these 2-DE maps, although prepared and analyzed non-consecutively, could be considered quite reproducible.

Proteins, whose antibodies were available in the laboratory (spots **a**, **e**, **g**, 1, 6, 9, 11, 12), were transferred onto PVDF membranes and incubated with the monoclonal antibodies indicated in the experimental section, followed by anti-rabbit antibody. Although not included among the altered proteins, we transferred also vimentin (expected to be under spot **z**). It was used as a sort of “internal standard” to obtain a further confirmation of the reproducibility of our map. The results of western blotting experiments are shown in Figure S3.

### Functional classification of differentially expressed proteins

Functional annotation of the altered proteins identified was carried out by categorizing these proteins into different groups based on Gene Ontology terms. As noted by GO, a significant proportion (around 50%) of these proteins were classified as having catalytic activity, in particular enzymes involved in metabolism of glucose. Also proteins involved in regulation were well-represented (around 40%). Cytoskeletal and transport proteins were present in small proportion (about 5% each).

## Discussion

Based on the similarity of clinical characteristics between Nasu-Hakola disease and other neuronal disorders, it cannot be expected that the former may be differentially diagnosed on the basis of patient’s symptoms only. In fact, failing the precise interpretation of NHD symptoms, patients are likely to go unrecognized and analyses at a molecular level are mandatory for a correct diagnosis of this pathology. In light of this, the identification of potential protein markers of the disease would indeed provide a novel context for a better understanding of the molecular mechanisms by which pathological events occur. Obviously, addressing this issue depends much on the ability to identify those proteins that are thought to be involved in these events. Under the assumption that changes in specific functional proteins play a key role in the pathogenesis of NHD, the objective of this study was thus to design a protocol aimed at identifying proteins that may be changed in this disease. 2-DE coupled to nano LC-MS/MS was the strategy that allowed, for the first time, to produce the proteomic profiles of lymphoblastoid B-cells from six individuals (four heterozygotes and two homozygotes) affected by this inherited rare disorder. By comparing protein patterns from these subjects with that of an healthy control of the same family, a number of spots which displayed altered expression could be observed. As by the graphical representation of spot variances shown in Fig. 3 (panels A and B), optical density of six spots (spots **a**; **f**; **h** and 1; 2 and 10 in figure 3) was increased in He and Ho compared to wt and that of eleven spots (spots **b**; **d**; **e**; **g** and 3; 4; 7–9; 12 and 13 in figure 3) was decreased. An oscillation pattern of changes (down-up-down or up-down-up) was observed for spots **c** and 6 and for spots 4 and 11, respectively.

The major question was, obviously, whether the change in expression of these proteins was in response to specific physiological conditions. If so, this was indeed an important proof of principle that the information contained in lymphoblastoid B-cells was able to reflect the health state of an organism.

### Proteins with catalytic activity

It should be emphasized that five, out of the 21 altered proteins identified in our study, were glycolytic enzymes and, what is more, they were the same enzymes indicated in previous proteomic studies as being somehow involved in neurodegenerative disorders different from NHD [29]–[34]. These included phosphoglycerate kinase 1 (PGK-1, spot 12); fructose bisphosphate aldolase A (aldolase A, spot **d**); phosphoglycerate mutase (PGM, spot **b**); glyceraldehyde-3-phosphate dehydrogenase (GAPDH, spot **e**) and α-enolase (spot **c**). Interestingly, in most cases, the expression level of these proteins in our patients (He and Ho) strongly correlated with the data of the literature for other neurodegenerative disorders [29]–[34].

For example, the proteomic profile obtained by Martinez *et al.* [29] from frontal cortex homogenates of patients with progressive supranuclear palsy (PSP), a neurodegenerative disorder characterized by neuronal loss and gliosis, resulted in the discovery of PGK-1 and aldolase A as targets of oxidation in oxidative stress. They proposed that the down-regulation of these enzymes might account for impaired energy metabolism in this disease. Being aldolase A (a glycolytic enzyme catalyzing the conversion of fructose bisphosphate into glyceraldehyde-3-phosphate dehydrogenase and dihydroxyacetone phosphate) present in neurons and astrocytes, and PGK-1 mainly in astrocytes, these findings support neurons and astrocytes as targets of oxidative damage in PSP. PGK-1 was described as being oxidized also in the frontal cortex of patients with *Alzheimer’s* disease (AD) [30], [31], and in transgenic mice with *Alzheimer* plaque pathology [32]. Experimental models of AD following injection of amyloid β_1–42_ peptide into rat brain [33] and in rat primary neural cells following amyloid β_1–42_–induced oxidative damage [34] have also evidenced the oxidation of several proteins related to glycolysis and glycogenesis. We can speculate that the evident down-regulation of PGK-1 (spot 12 in figure 3; 1.8-fold compared to control) and of Aldolase A (spot **d** in figure 3; 1.5-fold compared to control) in our patients may be responsible for impaired glucose metabolism resulting in the accumulation of glycolytic intermediates. Obviously these preliminary data do not allow to understand whether the role played by oxidative stress in the pathogenesis of NHD is similar to that demonstrated in other neurodegenerative disorders. Nevertheless, knowledge of the level of key proteins provides useful information for further investigations needed to clarify the mechanisms involved.

Likewise, also PGM (spot **b** in figure 3), an enzyme that catalyzes the interconversion of 3-phosphoglycerate and 2-phosphoglycerate in glycolysis and gluconeogenesis, was down-regulated (about 1. 6-fold compared to control) in He and Ho. Interestingly, PGM was among the proteins that were found to be under-expressed in a proteomic study performed on human brain (white and gray matter) from patients affected by corticobasal degeneration (CBD), an adult-onset progressive disorder [35]. Other proteins that were differentially expressed between CBD and non-demented comparison group included alcohol dehydrogenase; ubiquitin carboxyl-terminal hydrolase L1 (UCH L1) and L-isoaspartyl/D-aspartyl O-methyltransferase (PIMT) [35].

While not being glycolytic enzymes, UCH L1 and PIMT deserve our attention in this context. Emphasis should be placed, in particular, on UCH L1, one of the most abundant proteins in the brain, that was apparently down-regulated in CBD brain. By hydrolyzing a peptide bond at the C-terminal glycine of ubiquitin, this thiol protease is involved both in the processing of ubiquitin precursors and of ubiquitinated proteins. Given its function, UCH L1 was reported to be essential for brain function and required for normal synaptic and cognitive functions [36]. Down-regulation and oxidative modification of this enzyme were observed by other authors also in the brain of individuals with AD and Parkinson’s disease [30], [37]–[39]. More recently, a proteomic study revealed that the amount of UCH L1 was 2-fold decreased in hippocampus of zinc-deficient rats compared to controls [40]. This enzyme (spot 1 in figure 3) was found to be strongly up-regulated (about 3.2-fold compared to control) in our patients. This finding, while being in contrast with most data from the literature cited above, was in good agreement with the proteomic data obtained by Sultana *et* *al* [41] who determined, in hippocampus of AD patients, a 1.31-fold increase for this enzyme. These conflicting results could be tentatively explained by considering the time-course of NHD development. Although no evidence is currently supporting a direct relationship between UCH L1 and this neurodegenerative disorder, these changes provide new insights into the expression level of UCH L1 that could be promising in the search for sensitive and specific biomarkers of the disease.

L-isoaspartyl/D-aspartyl O-methyltransferase (PIMT) is a widely expressed protein-repair enzyme that restores isomerized or racemized aspartyl residues to their normal configuration. Generation of these residues was implicated in protein inactivation, autoimmunity and aggregation. In fact, it has been observed that the spontaneous formation in proteins (under physiological conditions) of atypical Asp residues (D-Asp and D, L-isoAsp) from L-Asp and L-Asn residues, can interfere with protein activity and lead to disruption of cellular function. Thus, the repair of atypical Asp residues by PIMT may function as a conformational switch in the regulation of cellular processes such as signal transduction [42]. Isomerized/racemized Asp residues have been shown to be increased in amyloid-beta (Aβ) peptides purified from the brain tissue of patients with *Alzheimer’s* disease (AD). Because isomerization/racemization of Aβ peptides enhances the aggregation process *in vitro,* this posttranslational modification is believed to be a pathogenic factor in the onset of sporadic cases of AD [42]. Working on mice, Yamamoto *et al.* [43] have also shown that deficiency of protein methylation leads to fatal progressive epileptic disease. Desrosiers and Fanelus [44] have reported that PIMT expression appears to decline during aging. The finding that PIMT is highly expressed in various stages of tissues (including embryonic and neonatal brains), suggests that this enzyme, in addition to the repair of aged proteins, may have roles in the brain and in other tissues. The down-regulation (around 2-fold compared to control) of this protein (spot 4 in figure 3) in He and Ho subjects of our study could reflect the changes that certainly take place in the brain (and/or in other tissues) of these individuals and we think it can be suggestive for the forthcoming studies of PIMT in NHD.

Glyceraldehyde-3-phosphate dehydrogenase (GAPDH) is a glycolytic enzyme with multiple functions, including a role as intracellular sensor of oxidative stress during early apoptosis. A large body of evidence, in fact, suggests that this enzyme is differentially affected *in vivo* in accordance with the degree of oxidative stress associated to neurodegenerative disorders. For example, abnormal expression and nuclear accumulation of this protein have been described in *postmortem* tissues from patients with several neurodegenerative diseases [45]. A decrease in protein level was found in brain of AD [41], [46] as well as in brain of transgenic mice [47] and in mice with amyotrophic lateral sclerosis [48]. Also in the frontal cortex of Lewy Body diseased patients [49] and in T-lymphocytes of Parkinson’s patients under dopaminergic therapies [50] the GAPDH levels were significantly different from those of controls. α-enolase, (the enzyme which interconverts 2-phosphoglycerate and phosphoenolpyruvate in glycolysis) is another target of oxidation in the frontal cortex. Its oxidation was described, among others, in patients with mild cognitive impairment and in advanced AD [41], [51]. The down-regulation of GAPDH (spot **e** in figure 3) in He and Ho (around 1.8-fold change compared to control) was in agreement with data from the literature and the oscillation pattern of changes of α-enolase (spot **c** in figure 3), although rather confusing (down-up-down), was coincident with results recently obtained by Takano *et al..* on AD model mice [52]. In fact, they observed that, while the amount of α-enolase was decreased in AD mice compared to controls, it significantly increased in the hippocampus of mice with amyloid deposition. Thus, the amyloid deposit was apparently responsible for the enhancement of the expression of energy metabolic proteins.

Taken together, all these data confirm, if necessary, that oxidative stress and damage are common molecular mechanisms at work in a variety of neurodegenerative disorders, including NHD. Nevertheless, it also appears that inhibition of glycolytic enzyme activities is a mere avenue by which these pathologies affect neuronal cell development and survival.

The amount of alcohol dehydrogenase (spot 11 in figure 3) was 1,1-fold higher in He and under-expressed in Ho (about 2.3-fold change compared to control). Numerous data in the literature [53]–[55] indicate that the interaction between amyloid-beta (Aβ) peptide-binding alcohol dehydrogenase (ABAD) and Aβ is an important mechanism involved in Aβ-mediated mitochondrial and neuronal perturbation. Inhibition of this interaction, in fact, was shown to significantly reduce mitochondrial Aβ accumulation [56]. Given its role, protection of the function of this specific target within the cell could be a route for preventing Aβ assemblies associated with synaptic failure and consequent mitochondrial dysfunction.

The up-regulation (about 3.60-fold change compared to control) of pyridoxal-5-phosphate phosphatase (PLPP) (spot f in figure 3), an enzyme that catalyzes the dephosphorylation of pyridoxal-5-phosphate, was in agreement with data obtained by Furukawa *et al.*. [57] on limbic forebrain of SAMP10 mouse, a model of age-related cerebral degeneration. Being PLPP one of the proteins known to be involved in brain cytoskeleton formation, and associated with acute and chronic neurodegenerative conditions, increased levels of this enzyme in their model were associated with aging. It has also been reported [58] that PLPP/chronophin-mediated actin dynamics may play an important role in the changes of morphological properties and excitability of the epileptic hippocampus.

### Cytoskeletal proteins and chaperones

Interaction of Aβ with β-actin, one of the major cytoskeletal proteins in neurons, was shown to enhance the neurotoxicity induced by tau-mediated actin filament formation. Moreover, as indicated above, the dynamics of β-actin assembly are involved in many aspects of cell motility, vesicle transport and membrane turnover. To elucidate the pathological effects of Aβ oligomers on hippocampus, Takano *et al.* [52] performed proteomic studies on AD model mice. Interestingly, while three out of the four spots containing β-actin showed a significant increase of this protein compared to controls, the level of the fourth was unchanged. By contrast, β-actin (spot **g** in figure 3) was found to be under-expressed (about 2.5-fold compared to control) in He and Ho of our study. This finding is of particular interest in the light of previous results obtained by Chen *et al.* [35]. Working on human brain from patients with CBD, they in fact observed an up-regulation of cofilin-1. However, being cofilins essential regulators of actin filament turnover, their increase implies an acceleration in actin filament depolymerization. Obviously, only the finding in He/Ho maps of over-expressed cofilin spot would confirm this hypothesis. Although the presence in our maps of additional β-actin spots cannot be excluded, the fact that they have not been selected by the statistical program means that no significant changes in intensity between control and patients could be observed.

The work of Takano *et al.* [52] also showed that Aβ oligomers might contribute to change the expression of heat shock protein 70 (Hsp70), a family of mammalian Hsps. These proteins not only work as chaperones to prevent protein misfolding and aggregation, but are also required to facilitate the transfer of misfolded proteins to proteasome for degradation [59]. In particular, the Hsp70 family includes both Hsc70 and Hsp70, the former being a cognate protein of the latter. Conflicting data are reported in the literature about the expression of Hsp70 in neurodegenerative disorders. In fact, while the above cited report of Takano *et al.* [52] described a significant decrease of this protein in hippocampus of AD model mouse, other studies indicate its up-regulation in hippocampus, inferior parietal lobe and cerebellum of subjects with mild cognitive impairment [60]. Elevated synthesis and accumulation of Hsp70 have been also observed in AD brain by Perez *et al..* [61]. Our results are in agreement with these data. The over-expression (about 2-fold compared to control) of Hsp70 (spot **a** in figure 3) in He and Ho maps seems to suggest an increased need of neural protection from stress by assisting cellular protein folding.

Other chaperon proteins have been found to be altered in NHD. In fact, chaperonin containing t-complex polypeptide 1 (TCP1) subunit ε (spot 2 in figure 3) and subunit γ (spot 7 in figure 3) were found to be up-regulated (around 3.2-fold compared to control) and down-regulated (about 4.6-fold compared to control), respectively. These data, if confirmed, may be of great interest. In fact, given that these chaperones have very similar roles in limiting the accumulation of misfolded proteins, one would have expected to observe the same behaviour for both subunits of TCP1. On the contrary, the expression level of subunit ε was strongly increased and that of subunit γ strongly decreased. Data of the literature appear contradictory. In fact, while TCP1 ε did not show any significant expressional change in AD brain [62], it was found increased in hippocampus of adolescent rats after excessive alcohol consumption [63]. On the other hand, proteomic analyses of S-nitrosylation of cysteine residues by NO has shown a large increase of TCP1 γ S-nitrosylation in neuroblastoma cells [64]. An imbalance of this process has also been linked to neurodegeneration through the impairment of pro-survival proteins [64].

### Other proteins

Elongation factor 1, subunit σ (spot 9 in figure 3), a protein that has been suggested to be implicated in the pathogenesis of neurodegenerative disorders, was found to be under-expressed (around 3-fold compared to control) in He and Ho of our study. Intriguingly, this protein was found to be up-regulated in mouse hippocampal HT22 cells treated with ochratoxin A (OTA) [65], a naturally occurring mycotoxin (produced by *Aspergillus ochraceus* and *Penicillum verrucosum*) that is found in a variety of plant food products such as cereals. The proteome response to OTA-induced cytotoxicity, included, among others, the alteration of elongation factor 1, subunit σ. Since reactive oxygen species (ROS) were detected in OTA-treated cells, the authors concluded that altered protein expression profile after OTA treatment was related to the generation of these species. In a recent report [66] it has been shown that elongation factor 1, subunit σ may inhibit *in vitro* and *in vivo* the activity of SIAH-1, an ubiquitin ligase, thus acting as a negative regulator of this activity. The possible role played by enoyl-CoA isomerase (spot 3 in figure 3) and coproporphyrinogen oxidase (spot 5 in figure 3) in NHD should be further investigated. The former is a mitochondrial enzyme involved in the degradation of unsaturated fatty acids by beta-oxidation [67]. Hydrophobic interactions between proteins and lipids or fatty acids have been well documented. It is known, in fact, that fatty acids have various effects on enzymatic activities of glycolytic enzymes [68]–[70]. The finding of this enzyme may add biochemical information on the involvement of metabolites with isoprenoid chain in NHD. Coproporphyrinogen oxidase is a key enzyme in heme biosynthesis whose partial enzymatic deficiency was found to be responsible for episodes of severe photosensitivity [71]. The former was under-expressed (around 1.8-fold changes compared to control) in He and Ho, while the latter had an up-down oscillation pattern of changes.

Heterogeneous nuclear ribonucleoprotein A1 is the best-known member of the hnRNPs family that has an important role in RNA metabolism and, by playing key roles in neuronal functioning and its depletion, is involved in several neurodegenerative disorders including AD; ALS; spinal muscular atrophy and fronto-temporal lobar degeneration [72]. The oscillation pattern of changes (down-up-down) did not allow us to have a clear picture of its real expression level in NHD patients. Given its importance, a precise determination of its expression level will constitute the core of future efforts.

Cystatins are cysteine-protease inhibitors implicated in various disease states, including neurodegenerative conditions [73]. It has been reported that dysregulation of cystatin B-cathepsin B signaling may serve as a critical mechanism coupling oxidative stress to neuronal degeneration in progressive myoclonus epilepsy [74]. Cystatin B (spot **h** in figure 3) was found to be up regulated (about 2.6-fold compared to control) in He and Ho. However, despite the belief that cystatin B is important for neurodegeneration, contrasting results have appeared in the literature and it is not clear, at the moment, whether low or high levels of cystatin B are beneficial for the brain [75].

Voltage-dependent anion-selective channel 1 (VDAC1) is one of the three isoforms of VDACs, known as mitochondrial porins. Together with isoform 2, VDAC1 forms pores in the biolipid layers of the mitochondrial outer membrane, thus being responsible for the characteristic permeability of this membrane [76]. Other important functions in the cell include regulation of calcium and ATP transport and of apoptosis signaling [77]–[79]. These functions have been found to be altered in cells from patients with neurodegenerative and mitochondrial diseases, leading to mitochondrial dysfunctions [80], [81] which have been identified as early events in AD pathogenesis, although their underlying mechanisms are not completely understood. Mitochondria dysfunction and oxidative stress have been extensively reported and the precise molecular link between mitochondrial dysfunction and AD pathogenesis was recently described [82]. Conflicting results have been reported in the literature concerning the level of VDAC 1 in human or mice brain tissues. In fact, the proteomic analysis of Yoo *et al.* [83] showed that total VDAC 1 was significantly decreased in frontal cortex and thalamus of post-mortem brain regions of patients with AD. By contrast, it was found over-expressed in the hippocampus of amyloidogenic AD transgenic mice models and in postmortem brain tissue from AD patients at an advanced stage of disease progression [84], [85].

In our study VDAC1 (spot 13 in figure 3) was under-expressed (about 1,6-fold change compared to control) in He and Ho. Emerging research has revealed that VDAC1 may be also found in the plasma membrane [86] in which it may represent a target for treatment of a range of conditions including neurodegenerative and mitochondrial diseases and possibly aging.

At this time the role of density-regulated protein (spot 8 in figure 3) and complex intermediate-associated protein 30 (spot 10 in figure 3) in NHD remains poorly understood. Nevertheless their identification may open new avenues for better understanding of mechanisms involved in the development of this disorder.

The complete list of up- and down-regulated proteins found in altered spots of our study, together with the values of their oscillation in expression level; their significance and the changes of the same proteins taken from the literature have been summarized in Table 3.

**Table 3:** Up- and down-regulated proteins found in altered spots of our study, together with the values of their oscillation in expression level.

Spot	Protein	Fold Change	Reference
	identified	(−/+)	
		wt *vs* He/wt *vs* Ho	
**a**	Heat Shock Protein −70	+1.44^a^/+2.5^a^	58,59
**b**	Phosphoglycerate mutase 1	−1.35^b^/−2.04^b^	33
**c**	α-enolase	+2.35^a^/+1.6^a^	50
**d**	Fructose bis-phosphate aldolase A	−1.55^b^/−1.54^b^	27–32
**e**	Glyceraldheyde-3 phosphate dehydrogenase	−1.75^b^/−1.83^b^	39; 44–48
**f**	Pyridoxal phosphate phosphatase	+2.80^a^/+4.42^a^	55
**g**	β-actin	−1.75^b^/−3.59^b^	33
**h**	Cystatin B	+2.22^a^/+3.12^a^	70
1	Ubiquitin carboxy-terminal Hydrolase L1	+3.18^a^/+3.31^a^	34–39
2	T-complex protein 1 subunit γ	+3.28^a^/+3,27^a^	60; 61
3	Δ(3,5)- Δ(2,4)-dienoyl CoA isomerase	−1.46^b^/−2.19^b^	67
4	L-isoaspartyl/D-aspartyl O-methyl transferase	−2.05^b^/−1.95^b^	41; 42
5	Coproporphyrinogen oxidase	+1.06^a^/−1.64^b^	71
6	Heterogeneous nuclear ribonucleoprotein A1	−1.43^b^/+1.49^a^	72
7	T-complex protein 1subunit ε	−2.88^b^/−6.54^b^	62
8	Density-regulated protein	−2.22^b^/−6.39^b^	–
9	Elongation factor 1 **δ**	−3.25^b^/−3.02^b^	63
10	Complex intermediate-associated protein 30	−3.03^b^/−2.61^b^	–
11	Alcohol dehydrogenaseNADP	+1.18^a^/−2.57^b^	51–54
12	Phosphoglicerate kinase 1	−1.39^b^/−2.27^b^	27–32
13	Voltage-dependent anion-selective channel protein 1	−1.40^b^/−1.74^b^	78–80

Reference numbers refer to reports previously published describing alterations of these proteins in different neurodegenerative diseases.

a p>0.05; ^b^p<0.05.

### Functional evaluation of data

**Figure 4:**
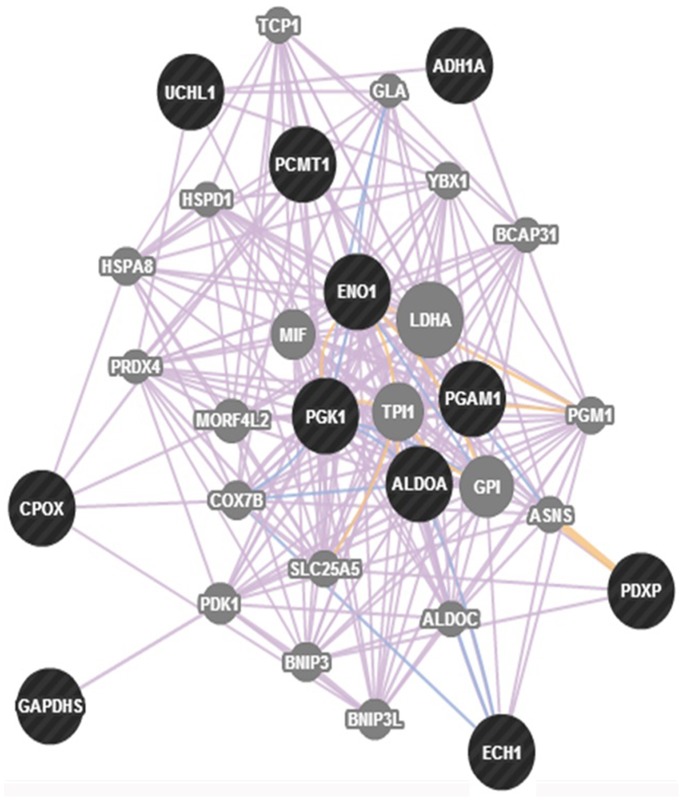
Gene network analysis obtained by navigating through the differentially expressed energy metabolic proteins identified in this study by using the GeneMANIA algorithm. Circles in black evidence genes encoding deregulated proteins used to generate the map and circles in grey evidence new genes that are functionally associated with the formers.

**Figure 5:**
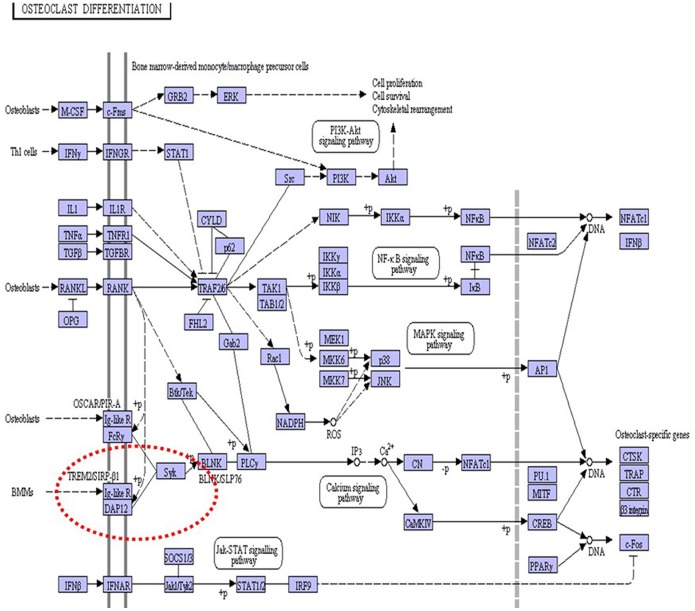
Osteoclast pathway map found in KEGG pathway database. The red dotted circle indicates the cascade activated by SYK.

The results discussed above, while providing a new and larger context for future studies on the pathogenesis of NHD, do not clarify yet whether the proteins observed to be differentially expressed between controls and patients are specific of NHD or not. A number of these proteins in fact intersects with a variety of neurodegenerative disorders, including AD; PD; ALS and others. In particular, the fact that several altered proteins are linked to glycolysis could support the idea that the general decrease of energy metabolism due to the reduced metabolic rate of glucose may be a feature of NHD, at least as far as the neurodegenerative aspect is concerned. Unfortunately the fact that, to maintain its functions, brain needs an enormous amount of energy compared with other tissues, is not such a surprising facet. In fact, that changes in these proteins may lead to major alterations in the energy pathways, thus affecting ATP production, was shown also for neurodegenerative diseases previously mentioned. In the light of our results it seems plausible to state that, in patients examined. the disturbed basal metabolic pathways, in the whole, are consistent with their previous, well-documented cognitive changes and clinical manifestations [17]. Thus, while clinical observations demonstrated abnormal cerebral cortex in these patients, the functional ones confirm that neurodegenerative processes extend beyond the basal ganglia. Obviously it remains largely a matter of speculation whether these glycolysis-related proteins contribute to the primary pathogenesis of the disorder, thus being specific biomarkers, or are a consequence of the disease process. Studies using *postmortem* brains of patients or microglia-like cells [87] in place of lymphoblastoid cells, might be a clue to understand better the biological basis of NHD. Nevertheless, in an effort to answer this question and to delineate the pathways these proteins could be involved in, the GeneMANIA algorithm (http://www.genemania.org) [88] was utilized in a function prediction setting. As shown in the gene map of Figure 4, new genes (circles in grey), that are functionally associated with those encoding deregulated proteins (circles in black) used to generate the map, were evidenced. From among these genes, at least three represent additional promising candidates involved in impaired glucose metabolism. We hypothesized that these proteins had not been identified in our study due to their low abundance. One of these is GPI that encodes a dimeric enzyme catalyzing the reversible isomerization of glucose-6-phosphate and fructose-6-phosphate. Outside the cell, this protein functions as a neurotrophic factor for spinal and sensory neurons. GPI deficiency can be associated with neurological impairment [89]. TPI 1 encodes a protein with a well-characterized role in glycolysis, catalyzing the isomerization of dihydroxyacetone phosphate to glyceraldehyde-3-phosphate. Although still poorly understood, a progressive neurodegenerative condition was shown to result from the deficiency of this enzyme [90]. The LDHA gene encodes lactate dehydrogenase-A which is a subunit of the lactate dehydrogenase enzyme. Being this enzyme important in providing energy for the body, its deficiency may determine a break-down in muscle tissue [91]. Finally, MIF gene encodes a key, regulatory cytokine which acts within both the innate and adaptive immune responses. Altered MIF regulation is considered important for acquiring chronic inflammation following an innate immune response. Recently, interest has increased in the role of MIF in the development of central nervous system tumors [92]. Moreover, giving GeneMANIA TREM2/DAP12 as query entry, the pathway in which these two genes are involved was also explored. From among the several genes which appeared to be in relation with to the two query genes, to make the research easier, only those genes which were well-integrated with the osteoclast pathway were selected. This approach led to the identification of a higher-order multimeric receptor complex containing TREM2, DAP12, plexin-A1 (PLXNA1) and semaphorine 6D (SEMA6D) [1]. In this complex, PLXNA1 was shown to act as co-receptor for SEMA6D, thus making the transmission of the signal to the membrane receptor TREM2 possible. DAP12 was shown to establish interesting physical and pathway interactions also with signal-regulatory protein beta 1 (SIRPB1), an immunoglobulin-like cell surface receptor that participates in the recruitment of SYK (spleen tyrosine kinase), a tyrosine kinase that activates a Ca^++^ cascade that leads to the nuclear gene activation [94]. SYK is an important player present in the cell cytoplasm that regulates different biological processes including innate and adaptive immunity, cellular adhesion, osteoclast maturation and vascular development. To activate the SYK protein, the ITAM (Immunoreceptor Tyrosine-based Activation Motif) tyrosine residues present on the DAP12 receptor must be phosphorylated by SRC family kinases. This step is followed by the recruitment and activation of SYK thanks to interaction between -SH2 domain on SYK and the ITAM domain on the receptor. By exploiting KEGG (Kyoto Encyclopedia of Genes and Genomes, http://www.kegg.jp/) Pathway, an osteoclast pathway map was found (see Fig. 5) from which it is easy to understand how the activation of SYK (evidenced by the red dotted circle) leads to a cascade that ends in the nucleus with the activation of specific osteoclast genes. All genes activated during this Ca^++^ dependent cascade are involved in bone remodeling, bone reabsorption and Ca^++^ homeostasis. In an effort to understand whether variations in the expression level of the 21 proteins that were observed to be over−/down-regulated during our study (HSPA4, PGAM1, ENO1, ALDOA, GAPDH, PDXP, ACTBL2, CSTB, UCHL1, CCT5, ECH1, PCMT1, CPOX, HNRNPH1, CCT3, DENR, EEF1D, NDUFAF1, AKR1A1, PGK1, VDAC2) could be related to a malfunction of the pathway, the genes that encode for these proteins were included in the osteoclast network made up of 14 genes (SYK, BLNK, PLCG1, PPP3CA, NFATC1, CTSK, ACP5, CALCR, ITGB3, CAMK4, CREB1, FOS plus TREM2 and DAP12). Given the difficulty to investigate the resulting network (not shown), due to the high number of genes inserted in the database, a number of genes that showed no/poor interactions were taken off from the pathway. As shown in Figure S3, the other genes that showed interactions with the osteoclast pathway genes, TREM-2 and DAP12, were maintained and integrated in the system. In particular, the proteins found in our study that seemed to participate in the system were: EEF1D, GAPDH, PGK1, HSPA4, AKR1A1, HNRNPH1, CCT5 and CCT3 (evidenced by a red-dotted line panel). With the aim to validate these results, the STRING (Search Tool for the Retrieval of Interacting Genes, http://string-db.org) database was applied indicating as query entry the 12 genes found to be involved in the osteoclast pathway plus TREM2 and DAP12 genes and the list of genes encoding the 21 altered proteins of this study (Figure S4). This database not only confirmed the data found with GeneMANIA but also added some new interactions to the system. As it can be seen in Figure S4, some new genes (in the red dotted-line circle) including PGAM1, VDAC2, ALDOA, ENO1 and PDXP resulted to be also well integrated in the osteoclast pathway. In particular 12 proteins (EEF1D, GAPDH, HSPA4, CCT3, CCT5, ENO1, PDXP, HNRNPH1, ALDOA, PGAM1, VDAC2 and PGK1), among the 21 proteins identified with the proteomic analysis, seem to be in relation with the osteoclast pathway. Even more interesting is the finding that about one half of these proteins (GAPDH, PGK1, PGAM1, ALDOA, ENO1) is involved in the glycolytic processes. Thus, a correlation between the genes involved in glycolysis and the genes involved in the osteoclast pathway does exist. It is, most likely, due to the big amount of ATP required from osteoclasts to perform correctly their function. Osteoclasts must generate sufficient ATP to carry on the energy-intense process of bone reabsorption. Bone is the only solid tissue in the human body and, as a dynamic tissue, is remodeled by a delicate balance between bone-forming osteoblasts and bone reabsorbing osteoclasts (95). Osteoclasts reabsorb bones by creating a subcellular compartment that is maintained at a low pH value, into which proteolytic enzymes are secreted. In this way the acidic environment removes the mineral phase. This function, together with the fact that osteoclasts are motile cells, represents an high demand of ATP (96). For this reason, as indicated by Kim *et al..* [95], metabolic pathways switch to an accelerated glycolytic and oxidative metabolism at an early stage of osteoclastogenesis. Another important evidence of the importance of glycolysis in osteoclasts was reported by Lu *et al.*. [97] who demonstrated that the E-subunit of the Vacuolar H^+^-ATPases binds the glycolytic enzyme aldolase and this binding may provide a basis for coupling glycolysis directly to the ATP-hydrolyzing proton pump. In osteoclasts, Vacuolar H^+^-ATPases (large multi-subunit proteins essential for acidification of intracellular compartments in eukaryotic cells) are densely packed in specialized domain of the apical plasma membrane where they acidify an extracellular compartment at the site of attachment to the bone thus playing an important role for a correct function of cells [98].

Under this scenario the importance of a functional glycolytic pathway during the osteoclastogenesis and the bone reabsorption becomes clearer. The fact that a mutation in the TREM2 or DAP12 gene can give rise to a non-functional membrane protein complex, can lead downstream to an altered intracellular pathway resulting in alteration of glycolytic gene expression. An altered glycolytic pathway that, as previously indicated, seems to be fundamental for the correct work of osteoclast cells, could be considered a key point to understand the osteodysplasia profile of Nasu-Hakola disease. This result can suggest a new field of investigation since, until now, there are no signs of proteomic research on the osteoclasts involved in this disease. Obviously, only performing proteomic analysis of the proteins expressed in osteoclasts it will be possible to validate this assumption.

### Limitation/strength of the study

The use of lymphoblastoid B-cells in place of micriglia as the source of our data may represent a limitation of this work. The question of whether results obtained from these cells could actually reflect possible dysfunction of microglia in Nasu-Hakola patients was the object of an intense debate inside the research team. Three factors did favor the use of lymphocytes. First, the finding of TREM2 expression in lymphoblastoid B-cells allowed to consider these cells suitable to identify molecules directly related to NHD. On the other hand, for the ethical considerations previously mentioned, microglia from living patients (and controls) involved in the research could not be available and surrogate cells had necessarily to be chosen for the study to be carried out. Second, lymphoblastoid B-cells provide continually sufficient biomaterial for proteomic analyses, which is not possible from direct specimen sampling of patients. The fact that re-sampling patients will not be required, while avoiding unnecessary discomfort to the patient, allays concerns of unavailable re-sampling because of patient geographical location, death or other factors. Third, the presence in the literature of a proteome map and a database of lymphoblastoid cells, generated by characterizing protein spots on 2-DE (26, 27), allowed to identify a number of spots by gel-matching. Thus, despite some inherent limitations, lymphoblastoid cell lines are increasingly recognized an important resource for genetic and functional research of neurological disorders. Nevertheless, in a very recent paper, Ohgidani *et al.* (87) have shown a novel technique aimed at developing directly induced microglia-like cells (iMG cells) with a combination of granulocyte-macrophage colony-stimulating factor (GM-CSF) and IL-34 from adult human monocytes without the use of viruses and genetic engineering. By comparing the levels of expression of TREM2 and DAP12 in iMG cells from a NHD patient and a healthy control, while no difference between them was evidenced for the former protein, the latter was significantly lower expressed in patient. Based on these results they hypothesized that iGM cells can possibly be utilized for analyzing the underlying microglial pathophysiology of brain disorders, although further investigations should be done to validate the closeness of iGM cells to human primary microglial cells in the brain.

A few words about the number of subjects investigated. It is probably fair to say that another limitation of the present study is the sample size of individuals investigated. We would like to note that the prevalence values of NHD in Europe (as by the Orphanet Report Series, Rare Disease Collection, dated November 2013) [99] is of 0.15/100.000. The prevalence in Japan and Finland, the two countries in which the majority of patients is confined, is of 0.2/100.000 and 1/100.000, respectively. The fact that no more than 200 cases have been identified worldwide, points to NHD as a very rare disease. As by a systematic survey of the literature, only three families (one of which decided to preserve its incognito) have been diagnosed for this disorder in Italy, for a total of about 10 to 12 people [100]. No information about possible additional sporadic cases is available. Thus, patients analyzed in this report, while being around 3% of total cases in the world, represent more than 50% of all Italian cases described so far. Moreover, to our knowledge, that involved in this study is the largest NHD family ever investigated and the only one for which genetic and radiological analyses of all components have been performed. In our opinion, the fact that all of patients originate from the same family, no matter how large the cohort is, makes this set of samples very uniform and represents a strength of the work although, of course, we are aware that a high-quality set of samples does not necessarily eliminate the risk of relying on poor evidence of data.

## Conclusions

Aim of this study was to identify protein biomarkers of NHD that could provide a novel context for facilitating interpretation of disease symptoms. This is the first attempt that gives just a taste of what is possible at the proteomic scale on NHD. While resulting in the identification of a good number of proteins differentially expressed between healthy controls and NHD patients, this pilot work has major limitations, first of all the tissue examined, that is lymphoblastoid cells. Proteins identified in these surrogate cells had been previously indicated as being involved in a variety of neurodegenerative disorders spanning from AD to PD, ALS and others. It could be argued that, being common to other brain disorders, these proteins were not very specific to NHD. However, given the similarity between clinical characteristics of NHD and those of other neuronal disorders, this finding was not such surprising. The experimental data reported here, while confirming that some relevant pathways shown to be involved in several brain disorders are, most likely, deregulated also in NHD, prove us right. Therefore, it cannot be expected that this pathology is differentially diagnosed on the basis of patient’s symptoms only. In terms of altered proteins identified and of their oscillation pattern of changes, the degree of agreement between our results and those previously published by other authors on neurodegenerative disorders different from NHD was even beyond our expectations. Such concordance of data is highly unlikely to be due to mere chance. Since, in most cases, also these authors used gel-based techniques (2-DE/MS) as the proteomic procedures to identify target proteins, this accordance may assess the analytical strength of techniques applied. In conclusion, our findings allow to speculate that changes in proteins of glycolisys and gluconeogenesis may lead to major alterations in the energy pathway metabolism which, on its turn, may explain puzzling symptoms of NHD patients, common to other brain disorders. In this context, these results may indeed represent a proof of principle for improving the knowledge of the disease. Aim of future studies will be understanding whether these alterations in glycolysis-related proteins are a cause or consequence of the disease process.

## Supporting Information

Figure S1 **PCR amplification of cDNA from Hela cells (lane 1, positive control); wild type homozygote II3 (lane 2); all patients considered in this study (lane 3→8) and 250 bp DNA ladder (lane 9).** The arrow indicates the position of TREM2.
(TIF)

Figure S2 **Western blotting on PVDF membrane of spots a, e, g, z (bottom, left to right) and spots 1, 6, 9, 11, 12 (top, left to right).**
(TIF)

Figure S3 **Analysis by GeneMANIA of the 35 genes inserted in the database.** The red dotted panel indicates the proteins found in our study that seemed to participate in the system.
(TIF)

Figure S4 **Analysis by STRING database with the 12 genes involved in the osteoclast pathway plus TREM2 and TYROBP genes and the list of 21 genes encoding proteins identified in our proteomic analysis.** The red dotted circle indicates five new genes which resulted to be well integrated in the osteoclast pathway.
(TIF)

Table S1 **Primary sequence of all peptides identified for each protein and data relative to their charge and molecular mass.**
(DOCX)

Table S2 **Primary sequence of all peptides identified for proteins a to h and data relative to their molecular mass and isoelectric points.**
(DOCX)
