# Supplementary figures and images for: Proteomic Analysis of Lymphoblastoid Cells from Nasu-Hakola Patients: A Step Forward in Our Understanding of This Neurodegenerative Disorder
Source: PLoS One. 2014 Dec 3;9(12):e110073. doi: 10.1371/journal.pone.0110073 (PMC4254282; doi:10.1371/journal.pone.0110073)

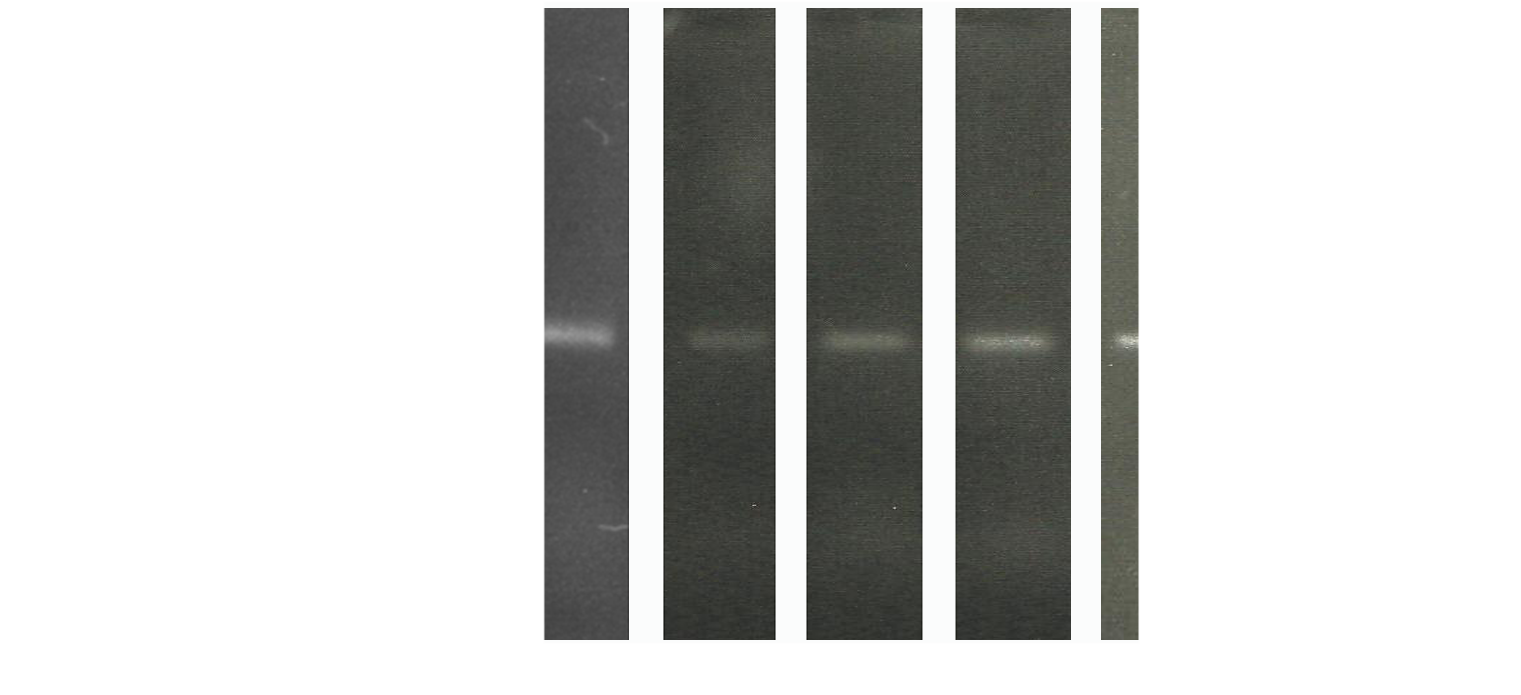

Supplement: Figure S1 — PCR amplification of cDNA from Hela cells (lane 1, positive control); wild type homozygote II3 (lane 2); all patients considered in this study (lane 3→8) and 250 bp DNA ladder (lane 9). The arrow indicates the position of TREM2. (TIF) [file pone.0110073.s001.tif]

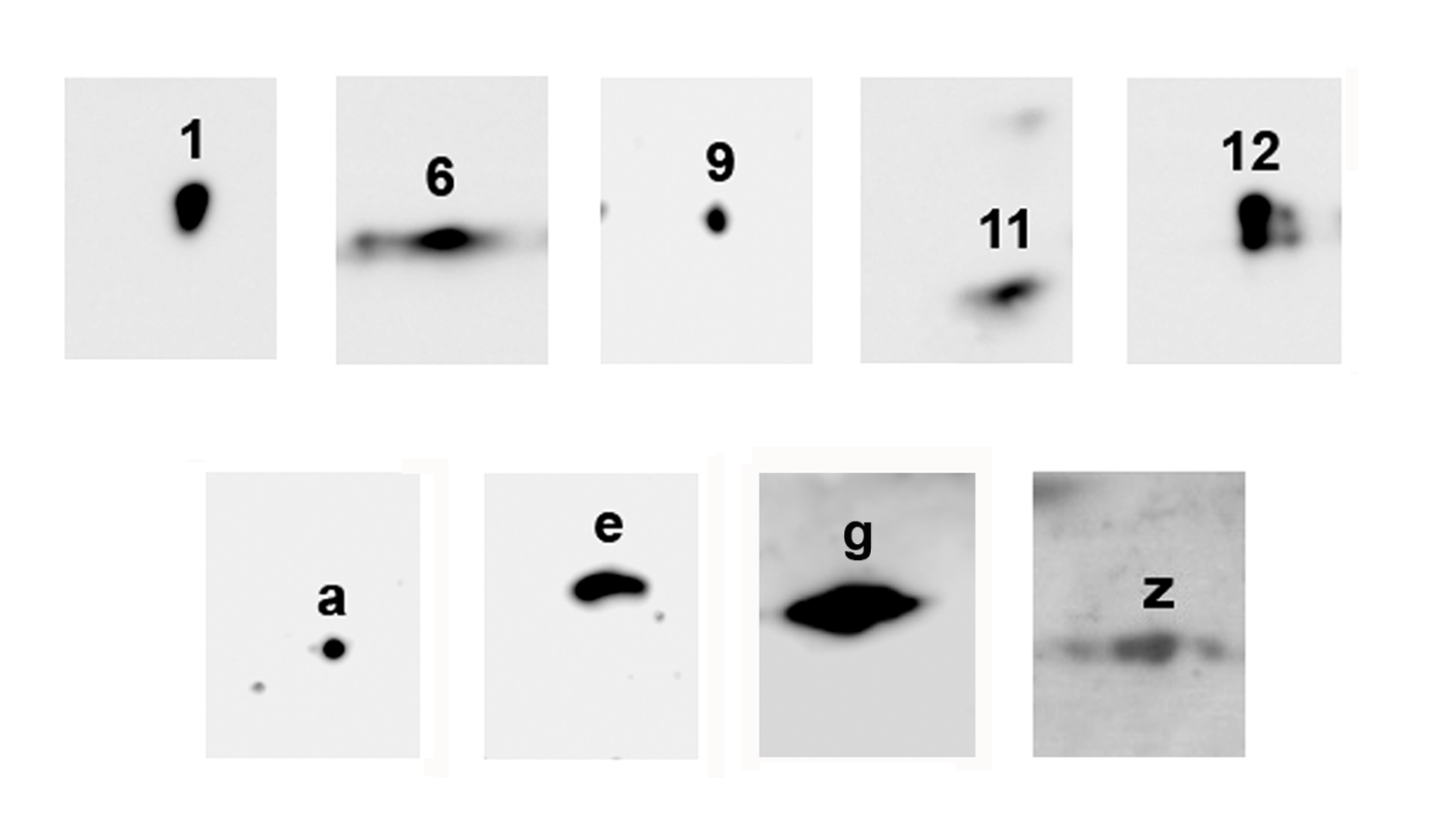

Supplement: Figure S2 — Western blotting on PVDF membrane of spots a, e, g, z (bottom, left to right) and spots 1, 6, 9, 11, 12 (top, left to right). (TIF) [file pone.0110073.s002.tif]

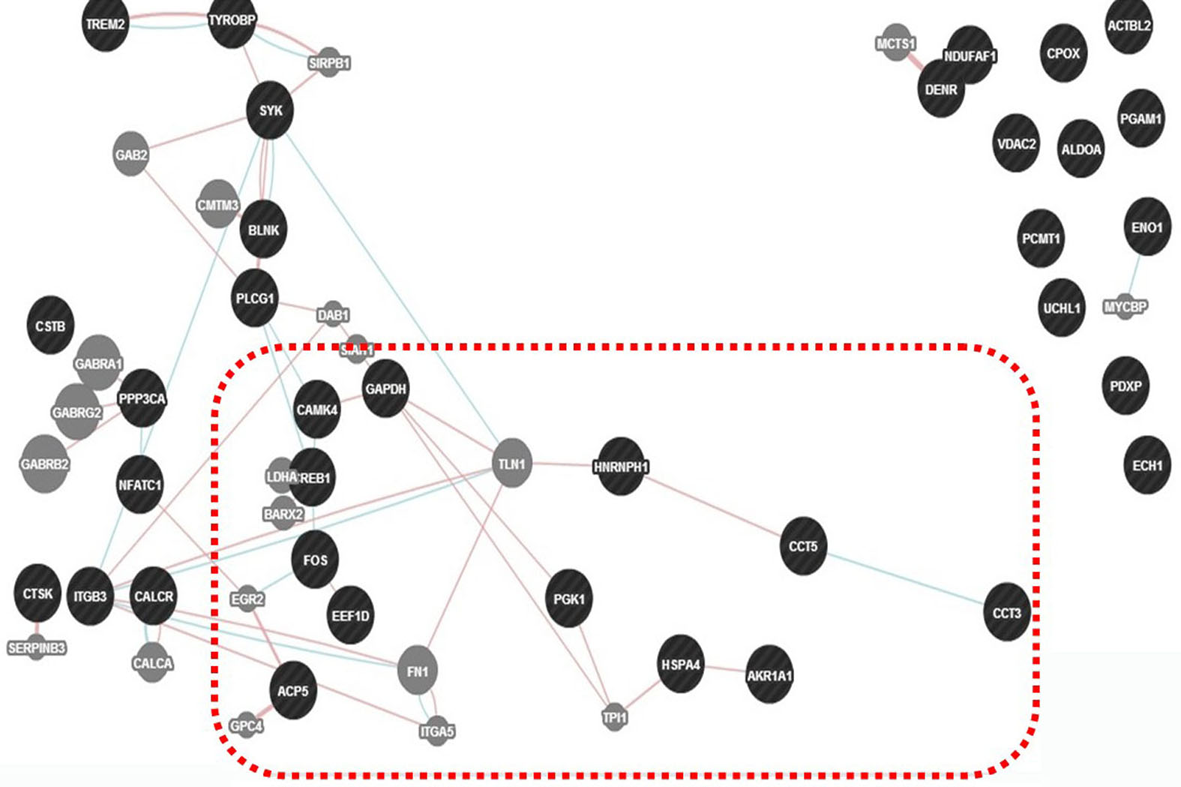

Supplement: Figure S3 — Analysis by GeneMANIA of the 35 genes inserted in the database. The red dotted panel indicates the proteins found in our study that seemed to participate in the system. (TIF) [file pone.0110073.s003.tif]

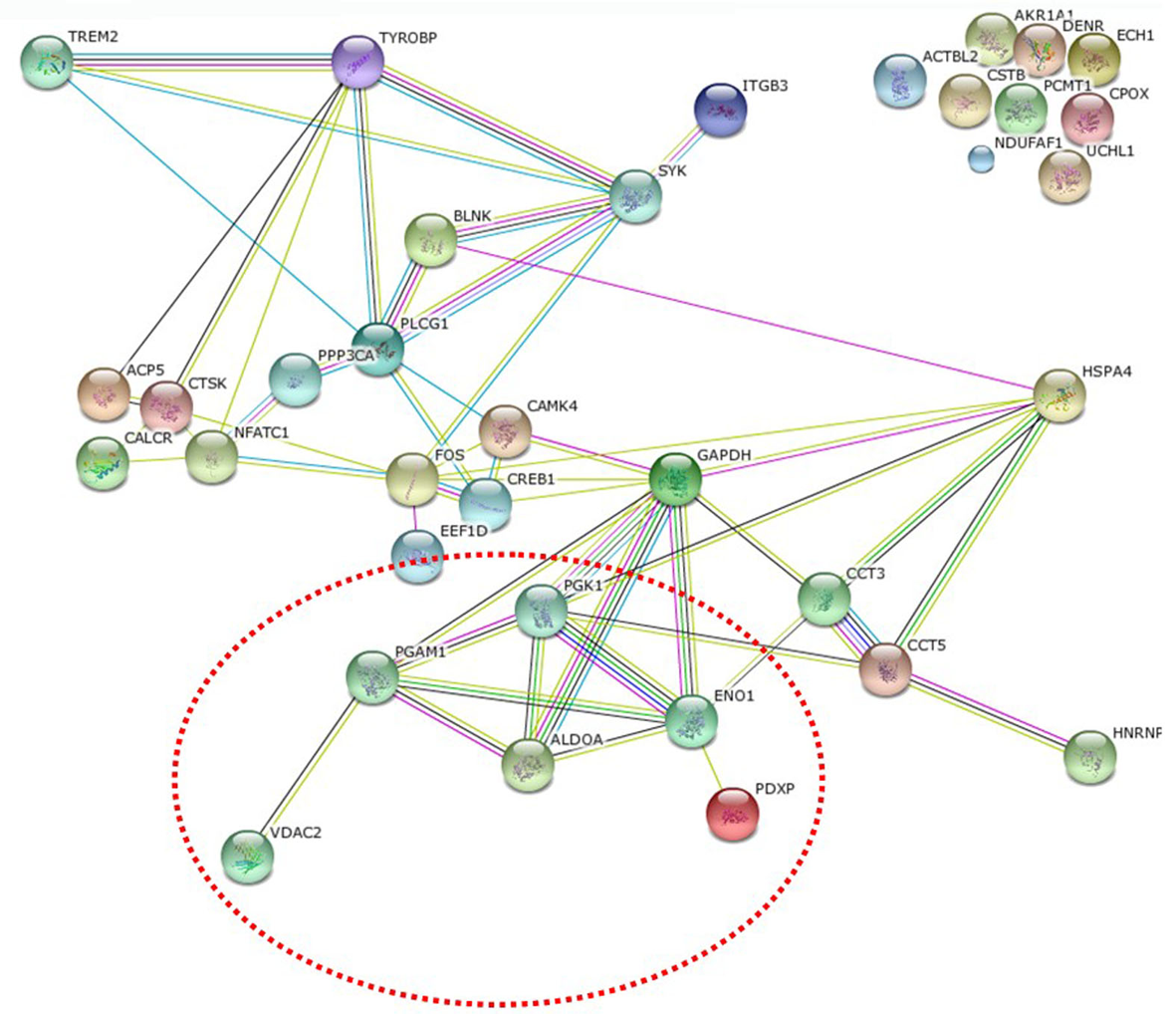

Supplement: Figure S4 — Analysis by STRING database with the 12 genes involved in the osteoclast pathway plus TREM2 and TYROBP genes and the list of 21 genes encoding proteins identified in our proteomic analysis. The red dotted circle indicates five new genes which resulted to be well integrated in the osteoclast pathway. (TIF) [file pone.0110073.s004.tif]
