# Supplementary material for: Proteomic Analysis of Lymphoblastoid Cells from Nasu-Hakola Patients: A Step Forward in Our Understanding of This Neurodegenerative Disorder
Source: PLoS One. 2014 Dec 3;9(12):e110073. doi: 10.1371/journal.pone.0110073 (PMC4254282; doi:10.1371/journal.pone.0110073)
Supplement: Table S1 — Primary sequence of all peptides identified for each protein and data relative to their charge and molecular mass. (DOCX) [file pone.0110073.s005.docx]

Table S1- Primary sequence of all peptides identified for each protein and data relative to their charge and molecular mass.

| **Spot No.** | **Protein name** | **Sequence** | **SpC** | **Protein Group Accessions** | **XCorr** | **Missed Cleavages** | **Charge** | **m/z**  **[Da]** | **MH+**  **[Da]** | **ΔM**  **[ppm]** | **Ions Matched** |
| --- | --- | --- | --- | --- | --- | --- | --- | --- | --- | --- | --- |
| **1** | Ubiquitin carboxy-terminal hydrolase L1 | MPFPVNHGASSEDTLLK | 9 | 4185720 | 6,094110489 | 0 | 2 | 921,9575806 | 1842,907885 | 1,266049612 | 25/32 |
|  |  | QIEELKGQEVSPK | 2 | 4185720 | 4,045740128 | 1 | 2 | 742,9016724 | 1484,796068 | 0,334595823 | 17/24 |
| **2** | T-complex protein 1 subunit ε | LGFAGLVQEISFGTTK | 6 | 194381764 | 4,296174526 | 0 | 2 | 834,4538574 | 1667,900438 | 0,034128986 | 19/30 |
|  |  | WVGGPEIELIAIATGGR | 6 | 194381764 | 3,950854778 | 0 | 2 | 869,9779053 | 1738,948534 | -0,125002621 | 20/32 |
| **3** | Δ(3,5)-Δ(2,4)-dienoyl-CoA isomerase, mitochondrial precursor | LTGSSAQEEASGVALGEAPDHSYESLR | 2 | 70995211 | 6,82464838 | 0 | 3 | 921,1016235 | 2761,290317 | -0,334606081 | 38/104 |
|  |  | VFPDKEVMLDAALALAAEISSK | 1 | 70995211 | 4,618873596 | 1 | 3 | 773,4156494 | 2318,232395 | 0,450210337 | 27/84 |
|  |  | EVMLDAALALAAEISSK | 3 | 70995211 | 4,218806744 | 0 | 2 | 866,4628906 | 1731,918505 | -0,76023785 | 18/32 |
|  |  | VIGNQSLVNELAFTAR | 1 | 70995211 | 3,886878014 | 0 | 2 | 866,4742432 | 1731,94121 | 1,338618258 | 14/30 |
|  |  | MMADEALGSGLVSR | 3 | 70995211 | 3,764146328 | 0 | 2 | 718,8475342 | 1436,687792 | 0,334407641 | 18/26 |
|  |  | EVDVGLAADVGTLQR | 1 | 70995211 | 3,626685619 | 0 | 2 | 771,9104614 | 1542,813646 | 0,871739478 | 17/28 |
| **4** | L-isoaspartyl/D-aspartyl O-methyltransferase | SGGASHSELIHNLR | 13 | 1718199 | 4,25147295 | 0 | 2 | 739,3786011 | 1477,749926 | -0,50466696 | 18/26 |
| **5** | Coproporphyrinogen oxidase | AGVSISVVHGNLSEEAAK | 18 | 433888 | 5,440056801 | 0 | 2 | 884,4658813 | 1767,924486 | 0,4778345 | 25/34 |
|  |  | ATSLGRPEEEEDELAHR | 18 | 433888 | 5,494138241 | 0 | 2 | 969,961853 | 1938,916429 | 0,597310266 | 24/32 |
|  |  | GIGGIFFDDLDSPSK | 7 | 433888 | 3,454945803 | 0 | 2 | 784,3862305 | 1567,765184 | 0,786480981 | 17/28 |
|  |  | GIGGIFFDDLDSPSKEEVFR | 3 | 433888 | 6,683371067 | 1 | 3 | 743,3688965 | 2228,092136 | 2,255249253 | 35/76 |
|  |  | IESILMSLPLTAR | 5 | 433888 | 3,276856661 | 0 | 2 | 722,4163818 | 1443,825487 | 1,001334225 | 19/24 |
|  |  | RGIGGIFFDDLDSPSK | 3 | 433888 | 4,361717701 | 1 | 2 | 862,4383545 | 1723,869432 | 2,529811303 | 22/30 |
|  |  | WEYMHSPSENSK | 3 | 433888 | 3,286800146 | 0 | 2 | 747,8207397 | 1494,634203 | 1,573342951 | 16/22 |
| **6** | Heterogeneous nuclear ribonucleoprotein H | ATENDIYNFFSPLNPVR | 4 | 48145673 | 4,067242146 | 0 | 2 | 998,9889526 | 1996,970629 | -2,885694201 | 17/32 |
|  |  | EGRPSGEAFVELESEDEVK | 2 | 48145673 | 5,251860142 | 0 | 3 | 702,9974976 | 2106,977939 | -2,260049396 | 26/72 |
|  |  | HTGPNSPDTANDGFVR | 9 | 48145673 | 4,650168419 | 0 | 2 | 842,8850708 | 1684,762865 | -2,728169366 | 25/30 |
|  |  | STGEAFVQFASQEIAEK | 4 | 48145673 | 3,885129452 | 0 | 2 | 921,447876 | 1841,888475 | -1,735157648 | 19/32 |
|  |  | VTGEADVEFATHEDAVAAMSK | 7 | 48145673 | 5,284060001 | 0 | 3 | 726,6713867 | 2177,999607 | -1,126902751 | 31/80 |
|  |  | YVELFLNSTAGASGGAYEHR | 11 | 48145673 | 5,602967262 | 0 | 3 | 714,6781616 | 2142,019932 | -2,427468877 | 32/76 |
| **7** | T-complex protein 1 subunit γ | IVSRPEELREDDVGTGAGLLEIK | 5 | 194374631 | 4,884447098 | 1 | 3 | 832,7826538 | 2496,333408 | 1,144428774 | 34/88 |
|  |  | KALDDMISTLK | 1 | 194374631 | 4,295410156 | 1 | 2 | 617,8400269 | 1234,672777 | 1,260083616 | 18/20 |
|  |  | KGESQTDIEITR | 5 | 194374631 | 4,066796303 | 1 | 2 | 688,8544922 | 1376,701708 | 0,033743693 | 21/22 |
|  |  | KGESQTDIEITREEDFTR | 3 | 194374631 | 4,084154129 | 2 | 3 | 718,6818237 | 2154,030918 | -0,052626261 | 30/68 |
| **8** | Density-regulated protein | QEAGISEGQGTAGEEEEK | 3 | 6048968 | 4,935947418 | 0 | 2 | 924,906311 | 1848,805345 | -2,231630116 | 23/34 |
|  |  | WPEVDDDSIEDLGEVK | 5 | 6048968 | 5,06268549 | 0 | 2 | 923,4219971 | 1845,836718 | -1,226424501 | 24/30 |
|  |  | WPEVDDDSIEDLGEVKK | 4 | 6048968 | 5,123298168 | 1 | 3 | 658,6488037 | 1973,931858 | -1,060531639 | 31/64 |
| **9** | Elongation factor 1 δ | SLAGSSGPGASSGTSGDHGELVVR | 5 | 119602640 | 5,682966709 | 0 | 3 | 729,0202026 | 2185,046055 | -0,932080969 | 38/92 |
| **10** | Complex intermediate-associated protein 30, mitochondrial | GPEGHPLHEVLLEQAK | 2 | 4929599 | 5,493637085 | 0 | 3 | 585,3087158 | 1753,911594 | -6,640636336 | 30/60 |
| **11** | Alcohol dehydrogenase[NADP(+)] | AWRDPDEPVLLEEPVVLALAEK | 5 | 119627378 | 6,101629734 | 1 | 3 | 830,4414673 | 2489,309849 | -7,597565462 | 36/84 |
|  |  | DPDEPVLLEEPVVLALAEK | 38 | 119627378 | 4,93721056 | 0 | 2 | 1038,553589 | 2076,099901 | -5,442987411 | 21/36 |
|  |  | GLEVTAYSPLGSSDR | 6 | 119627378 | 3,63215518 | 0 | 2 | 776,3807373 | 1551,754198 | -6,955554326 | 19/28 |
|  |  | GLVQALGLSNFNSR | 1 | 119627378 | 3,472764969 | 0 | 2 | 738,3967896 | 1475,786302 | -6,958201482 | 15/26 |
| **12** | Phosphoglycerate kinase 1 | AHSSMVGVNLPQK | 1 | 194384036 | 4,34967947 | 0 | 2 | 684,3529663 | 1367,698656 | -8,339046589 | 19/24 |
|  |  | ALESPERPFLAILGGAK | 5 | 194384036 | 5,582102299 | 0 | 2 | 884,9968872 | 1768,986498 | -5,202699935 | 23/32 |
|  |  | ITLPVDFVTADKFDENAK | 3 | 194384036 | 4,903597355 | 1 | 3 | 675,0131836 | 2023,024998 | -6,600891618 | 27/68 |
|  |  | LGDVYVNDAFGTAHR | 1 | 194384036 | 3,93035531 | 0 | 2 | 817,8929443 | 1634,778612 | -8,31870935 | 20/28 |
|  |  | QIVWNGPVGVFEWEAFAR | 2 | 194384036 | 4,044948101 | 0 | 2 | 1053,026001 | 2105,044725 | -7,461108396 | 19/34 |
|  |  | VSHVSTGGGASLELLEGK | 2 | 194384036 | 5,690379143 | 0 | 2 | 870,9525757 | 1740,897875 | -8,539589271 | 28/34 |
| **13** | Voltage -dependent anion-selective channel protein 2 | TGDFQLHTNVNDGTEFGGSIYQK | 2 | 194383758 | 5,877930641 | 0 | 3 | 843,3886719 | 2528,151462 | -6,901851119 | 29/88 |
|  |  | VNNSSLIGVGYTQTLRPGVK | 5 | 194383758 | 5,230484009 | 0 | 2 | 1052,073853 | 2103,140428 | -7,290450827 | 21/38 |
