# Supplementary material for: Proteomic Analysis of Lymphoblastoid Cells from Nasu-Hakola Patients: A Step Forward in Our Understanding of This Neurodegenerative Disorder
Source: PLoS One. 2014 Dec 3;9(12):e110073. doi: 10.1371/journal.pone.0110073 (PMC4254282; doi:10.1371/journal.pone.0110073)
Supplement: Table S2 — Primary sequence of all peptides identified for proteins a to h and data relative to their molecular mass and isoelectric points. (DOCX) [file pone.0110073.s006.docx]

Table S2- Primary sequence of all peptides identified for proteins **a** to **h** and data relative to their molecular mass and isoelectric points.

| **Spot** | **Protein Name** | **Gene** | **Accession Number** | **pI** | **Mass**  **kDa** | **Score (%)** | **Peptides** |
| --- | --- | --- | --- | --- | --- | --- | --- |
| **a** | Stress-70 protein, mitochondrial | **HSPA9** | sp\|P38646\|GRP75 | 5.9 | 73.681 | 88 | HQDSWNGLSHEAFR |
|  |  |  |  |  |  |  | RTIAPCQK |
|  |  |  |  |  |  |  | DIKNVPFK |
|  |  |  |  |  |  |  | VQQTVQDLFGRAPSK |
|  |  |  |  |  |  |  | MISASR |
| **b** | Phosphoglycerate mutase 1 | PGAM1 | sp\|P18669\|PGAM1_HUMAN | 6.7 | 28.804 | 91 | ALPFWNEEIVPQIK |
|  |  |  |  |  |  |  | DRRYADLTEDQLPSCESLK |
|  |  |  |  |  |  |  | AETAAKHGEAQVKIWR |
| **c** | Alpha-enolase | **ENO1** | sp\|P06733\|ENOA | 5.9 | 47.169 | 90 | TGAPCRSER |
|  |  |  |  |  |  |  | FGANAILGVSLAVCK |
|  |  |  |  |  |  |  | NFRNPLAK |
| **d** | Fructose-bisphosphate aldolase A | **ALDOA** | sp\|P04075\|ALDOA | 8.4 | 39.421 | 99 | ALQASALK |
|  |  |  |  |  |  |  | QLLLTADDR |
|  |  |  |  |  |  |  | GILAADESTGSIAK |
|  |  |  |  |  |  |  | GVVPLAGTNGETTTQGLDGLSER |
| **e** | Glyceraldehyde-3-phosphate dehydrogenase | **GAPDH** | sp\|P04406\|G3P | 8.6 | 36.053 | 97 | GALQNIIPASTGAAK |
|  |  |  |  |  |  |  | LISWYDNEFGYSNR |
|  |  |  |  |  |  |  | VKVGVNGFGRIGRLVTRAAFNSGK |
| **f** | Pyridoxal phosphate phosphatase | **PDXP** | sp\|Q96GD0\|PLPP_HUMAN | 6.1 | 31.698 | 89 | VRAVLVGYDEHFSFAK |
|  |  |  |  |  |  |  | LARAGKAALFVSNNSR |
|  |  |  |  |  |  |  | MARCERLRGAALR |
| **g** | β-actin | ACTB | sp\|P60709\|ACTB_HUMAN | 5.3 | 41.737 | 93 | AGFAGDDAPR |
|  |  |  |  |  |  |  | MDDDIAALVVDNGSGMCK |
|  |  |  |  |  |  |  | IIAPPER |
| **h** | Cystatin-B | CSTB | sp\|P04080\|CYTB_HUMAN | 6.9 | 11.143 | 90 | ENKKFPVFKAVSFK |
|  |  |  |  |  |  |  | VFQSLPHENKPLTLSNYQTNKAK |
